# *N*-Hydroxyethyl acrylamide as a functional eROP initiator for the preparation of nanoparticles under “greener” reaction conditions†

**DOI:** 10.1039/d2py00849a

**Published:** 2022-10-10

**Authors:** Joachim C. Lentz, Robert Cavanagh, Cara Moloney, Bruno Falcone Pin, Kristoffer Kortsen, Harriet R. Fowler, Philippa L. Jacob, Eduards Krumins, Charlotte Clark, Fabricio Machado, Nicholas Breitkreuz, Ben Cale, Amy R. Goddard, Jonathan D. Hirst, Vincenzo Taresco, Steven M. Howdle

**Affiliations:** School of Chemistry, University of Nottingham, University Park NG7 2RD Nottingham UK vincenzo.taresco@nottingham.ac.uk steve.howdle@nottingham.ac.uk; School of Pharmacy, University of Nottingham, University Park NG7 2RD Nottingham UK; Croda Europe Limited Cowick Hall Snaith DN14 9AA Goole UK; Institute of Chemistry, University of Brasília Campus Universitário Darcy Ribeiro 70910-900 Brasília DF Brazil

## Abstract

*N*-Hydroxyethyl acrylamide was used as a functional initiator for the enzymatic ring-opening polymerisation of ε-caprolactone and δ-valerolactone. *N*-Hydroxyethyl acrylamide was found not to undergo self-reaction in the presence of Lipase B from *Candida antarctica* under the reaction conditions employed. By contrast, this is a major problem for 2-hydroxyethyl methacrylate and 2-hydroxyethyl acrylate which both show significant transesterification issues leading to unwanted branching and cross-linking. Surprisingly, *N*-hydroxyethyl acrylamide did not react fully during enzymatic ring-opening polymerisation. Computational docking studies helped us understand that the initiated polymer chains have a higher affinity for the enzyme active site than the initiator alone, leading to polymer propagation proceeding at a faster rate than polymer initiation leading to incomplete initiator consumption. Hydroxyl end group fidelity was confirmed by organocatalytic chain extension with lactide. *N*-Hydroxyethyl acrylamide initiated polycaprolactones were free-radical copolymerised with PEGMA to produce a small set of amphiphilic copolymers. The amphiphilic polymers were shown to self-assemble into nanoparticles, and to display low cytotoxicity in 2D *in vitro* experiments. To increase the green credentials of the synthetic strategies, all reactions were carried out in 2-methyl tetrahydrofuran, a solvent derived from renewable resources and an alternative for the more traditionally used fossil-based solvents tetrahydrofuran, dichloromethane, and toluene.

## Abstract 2

*N*-Hydroxyethyl acrylamide was used as a functional initiator to produce hybrid macromonomers *via* the enzymatic ring-opening polymerisation of ε-caprolactone and δ-valerolactone.

## Graphical abstract

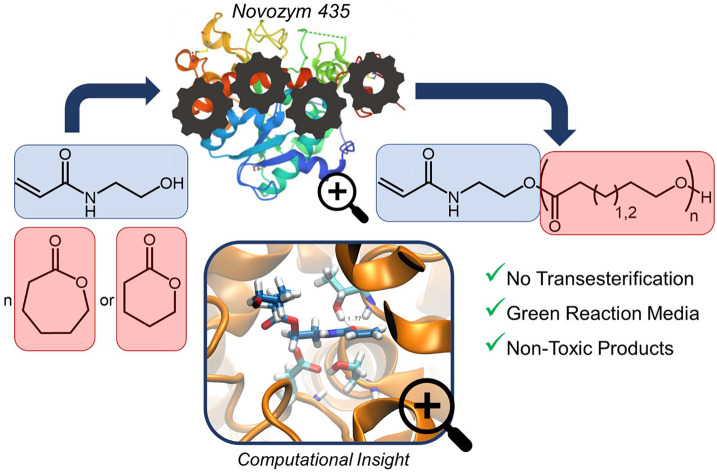

## Introduction

Aliphatic polyesters such as poly(caprolactone) (PCL), poly(lactic acid) (PLA), and poly(lactic-*co*-glycolic acid) have gained significant interest because of their biodegradability and biocompatibility.^1^ These properties, in combination with their low cost of production, have made this class of polymer especially interesting for pharmaceutical applications.^2^ To increase the scope of application, terminal and side-chain functional groups can be installed.^3^ The introduction of a reactive double bond to the polymer enables the synthesis of functional polyesters, that can be reacted further or post-polymerised to suit the target application.^1,4–6^

Ring-opening polymerisation (ROP) allows for the synthesis of aliphatic polyesters through cyclic monomers such as lactide, lactones (*e.g.* caprolactone, β-propiolactone, γ-butyrolactone, δ-valerolactone, ε-caprolactone), among others.^3^ Relief of ring-strain rather than removal of condensate allows ROP to take place under milder conditions than those required for polycondensation.^7^ Milder conditions facilitate the use of thermally sensitive monomers as well as limiting discolouration and degradation during synthesis.^8^ Furthermore, by addition of an initiator in ROP, a variety of end-groups can be installed.^9^ The presence of an initiator also allows control over targeted molecular weight by varying the ratio of initiator to monomer.^10^ Functional ROP initiators provide a straightforward pathway to produce aliphatic polyesters that can be further derivatized post-polymerisation. 2-Hydroxyethyl methacrylate (HEMA) is a well-studied example of one such functional initiator, since it contains both a primary hydroxyl and a methacrylic group.^1,5,6,11–13^ HEMA has been used as ROP initiator for enzymatic, metal-based, and organocatalytic routes.^1,14^

A large portion of published research on these functional ROP systems features tin(ii) 2-ethylhexanoate as catalyst.^2,3^ Drawbacks of these systems include difficulty of removing tin residues during purification; particularly important for biomedical devices.^1,15,16^ Furthermore, these catalysts exhibit high toxicity towards mouse fibroblasts and human endothelial cells fibroblasts.^17^

Enzymatic catalysis is a viable metal-free alternative to produce polymers by ROP (eROP).^3,15,18^ Using an enzyme that is both commercially available and immobilized overcomes a plethora of issues traditionally associated with enzymatic and organo-catalysis.^19^ Commercial availability removes the need for in-house enzyme purification and production expertise. Enzyme immobilization can be used to improve stability, activity, and selectivity.^19,20^ In this context however, the improved chemical resistance, recyclability, and ease of handling are the greatest benefits of immobilisation.^19,20^ Lipase B from *Candida antarctica* (CALB), which has been extensively studied for eROP and enzymatic polycondensation, is commercially available in an immobilized formulation under the name Novozym 435 (N435).^3,15,21^

Novozym 435 catalysed ROP, using HEMA or other ester containing molecules as ROP initiators, has been limited by a lack of differentiation between the ester bond in the initiator and the ester bond in the monomer.^5^ Further radical polymerisation techniques are made impossible by transesterification, since the ethylene glycol dimethacrylate that is formed will act as a cross-linker yielding insoluble material. In addition, the ethylene glycol, produced as a by-product, can act as an eROP initiator – producing chains lacking the desired end group functionality (ESI Fig. 1†). The lack of selectivity leads to methacrylate and polyester transfers in eROP.^5,6,22^ Takwa *et al.*^5^ used HEMA initiated eROP followed by addition of vinyl methacrylate to create a series of biodegradable cross-linkers. However, for the synthesis of unbranched polymers, transesterification severely limits the use of acrylates as initiators in eROP. To initiate eROP whilst maintaining control over the end-groups, it is imperative that the initiator is stable in the presence of the enzyme.

**Fig. 1:**
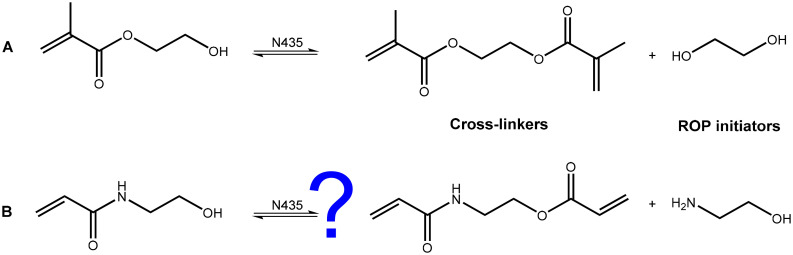
A: transesterification of HEMA in the presence of N435 leads to cross-linkers and diols capable of acting as bifunctional eROP initiators which lead to the formation of mono-methacrylate, di-methacrylate, and non-methacrylated poly(caprolactone) products, seen in ESI Fig. 1.† B: HEAA however, is hypothesised not to undergo analogous reactions in the presence of N435.

To overcome issues of transesterification associated with methacrylate and acrylate based initiators, in the present work, we considered acrylamides as alternative ROP initiators. Since the amide bond exhibits greater stability towards nucleophilic attack, it was thought that a serine-based lipase such as CaLB relying on an initial nucleophilic attack would be unable to catalyse self-reaction (Fig. 1B) in the working conditions employed.^3^*N*-Hydroxyethyl acrylamide (HEAA) was identified as a potential alternative to HEMA due to its analogous structure, low cost, and commercial availability. Like HEMA, HEAA contains both a primary hydroxyl as well as a radically polymerisable group. HEAA has been widely utilized in coatings, sealants, thermosetting paints, and personal care products.^23^ Poly(HEAA) has strong anti-fouling properties,^24^ and has received particular interest as a material for protein separation,^25^ and drug delivery systems.^26,27^

**Fig. 2:**
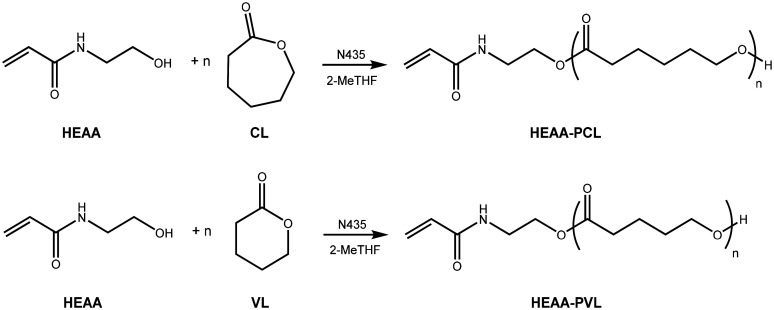
Targeted reactions of HEAA initiated eROP of caprolactone and valerolactone.

Motivated by the properties of HEAA that make its use in biomaterials advantageous, this paper investigates the use of HEAA as a functional eROP initiator for nanoparticle (NP) formulation. The stability of HEAA was confirmed in the presence of N435, and so HEAA was investigated as an eROP initiator for both caprolactone (CL) and valerolactone (VL), according to the reaction scheme show in Fig. 2.

Both HEAA-PCL and HEAA-PVL contain an acrylamide end-group, which is in principle available for radical polymerisation post eROP. Radical co-polymerisation can be used to tune hydrophilicity by introduction of water-soluble co-monomers. Tuning the hydrophilicity should allow control over the size and stability of NPs produced. The presence of a degradable aliphatic polyester should also allow excretion of the NPs, thereby avoiding bioaccumulation.^28^

Dichloromethane (DCM) and tetrahydrofuran (THF) are solvents routinely used for a variety of polymerisation processes, owing to their excellent solubility parameters, they are unfortunately derived from petrochemical feedstocks. DCM is also known to be carcinogenic and contribute to ozone layer depletion.^29^ In order to avoid using petroleum derived chemicals alternatives are required. 2-Methyl tetrahydrofuran (2-MeTHF) is a bio-based solvent, produced from chemical and enzymatic treatment of biomass, and has similar solubility parameters to petrochemical solvents.^30–32^ In addition, it possesses a relatively high boiling point (80 °C), and a lower critical solution temperature with water than THF – crucial for limiting hydrolytic initiation of ROP. Furthermore, the cost of 2-MeTHF on a lab-scale is comparable to that of non-renewable solvents. Previous work in the group has demonstrated the suitability of 2-MeTHF as a solvent for multi-polymerisations combining eROP with both free-radical and reversible addition–fragmentation chain transfer (RAFT) polymerisation.^4^

In this paper we report the use of HEAA as a functional initiator in eROP, and subsequent Free-Radical Polymerisation (FRP) of the produced macromonomers. HEAA initiated eROP was studied in-depth by ^1^H-NMR spectroscopy and incomplete initiator conversion was observed. Computational docking studies were employed to gain insight into the cause of incomplete initiator conversion. End-group fidelity of the materials was demonstrated by chain extension using the organocatalyzed ROP of lactide, as well as radical (co-)polymerisation. The materials were shown to be non-toxic through *in vitro* tests against lung, intestinal, and skin cells. Amphiphilic co-polymers of HEAA-PCL and PEGMA were assembled into polymeric NPs by nanoprecipitation.

## Results and discussion

### Stability of HEAA towards N435

Because of the structural similarity of HEAA and HEMA, it was important to ensure HEAA does not exhibit reactions analogous to the transesterification reactions observed with HEMA (Fig. 1).^6^ The lability of HEAA in the presence of N435 was screened. HEMA was used as a positive control, as it is well-known to undergo transesterification with itself in the presence of N435 to form ethylene glycol dimethacrylate and ethylene glycol (Fig. 1A). From ^1^H-NMR analysis, it is clearly seen that HEMA undergoes transesterification (ESI Fig. 2†), confirming previous observations,^33^ while HEAA remains unchanged when subjected to reaction conditions (ESI Fig. 3†). Transesterification of HEMA can be detected from the appearance of peaks assigned to ethylene glycol and ethylene glycol dimethacrylate.^33^ The formation of ethylene glycol dimethacrylate results in the vinyl peaks splitting and allows straightforward calculation of the extent of transesterification. On the other hand, in the case of HEAA no peaks corresponding to ethanolamine (triplets at 3.58 and 2.81 ppm, ESI Fig. 4†) are observed in the ^1^H-NMR spectrum (ESI Fig. 3†), indicating that HEAA is stable under reaction conditions (65 °C, and 20 h of contact) in the presence of N435. Various N435 loadings were screened (5, 10, and 20 wt%, Table 1). No change was detectable in the case of HEAA, while transesterification of HEMA increased up to 41% using a 20 wt% N435 loading.

**Table 1:** Various N435 loadings were screened to assess the stability of HEAA. HEMA is included as a positive control as it is known to react with itself in the presence of N435

N435 loading (wt% w.r.t initiator)	Transesterification of HEMA after 20 h^a^ (%)	Self-reaction of HEAA after 20 h^a^ (%)
5	25	Not detectable
10	29	Not detectable
20	41	Not detectable

a Determined by ^1^H-NMR.

It should be noted that HEAA, unlike HEMA, is associated with Hazard Statement H373 “May cause organ damage through prolonged or repeated exposure”. This is for the monomers. However once polymerised, neither the eROP synthesised macroinitiator nor the related FRP synthesised homo and (co-)polymers showed any relevant cytotoxicity in the *in vitro* experiments performed (see Cytotoxicity measurements Fig. 10).

The lack of reactivity of HEAA towards itself in the presence of N435 is a strong indication that the amide bond in HEAA is stable under the reaction conditions employed during eROP. The extra stability of HEAA over HEMA can be explained by the lower electronegativity of nitrogen compared to that of oxygen, leading to greater delocalisation of the nitrogen lone pair into the carbonyl bond.^34,35^ As a result, the carbonyl oxygen resonance hybrid already holds a more significant partial negative charge decreasing its susceptibility towards nucleophilic attack.

*Candida antarctica* Lipase B (CALB), the active enzyme in N435, catalyses esterification *via* an initial nucleophilic attack of the carbonyl bond by an activated serine residue in its active site.^36^ It was hypothesised that the initial nucleophilic attack of the catalytic cycle might be inhibited by the decrease in electrophilicity going from an ester to an amide (ESI Fig. 5†).

The stability of the initiator is vital to enable synthesis of HEAA initiated ROP products whilst maintaining control over the end groups. The promising results (ESI Fig. 3 and Table 1†) gave us confidence that HEAA is suitable as an eROP initiator, and CL and VL were selected to be the model monomers for HEAA initiated eROP using N435.

### HEAA initiated eROP in 2-MeTHF of ε-caprolactone and δ-valerolactone

#### Lactone conversion

**Fig. 3:**
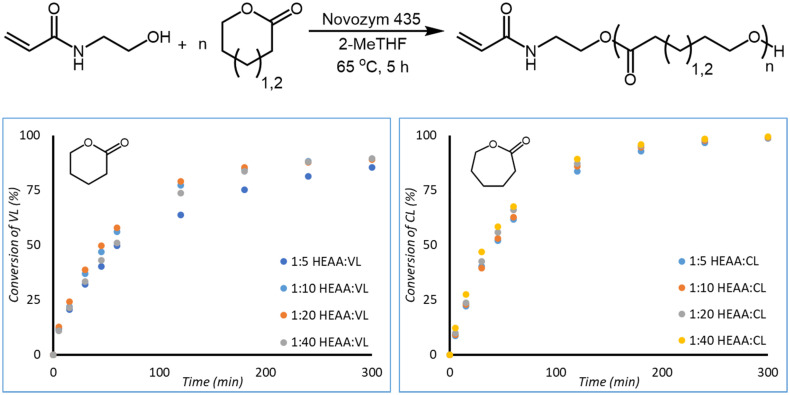
Reaction scheme of HEAA initiated eROP of CL and VL (top). Conversion of VL (bottom left) and CL (bottom right) over time for the HEAA initiated eROP in 2-MeTHF and 10 wt% N435. Reactions were performed at a monomer concentration of 0.143 g mL^−1^.

Reactions using HEAA as a functional eROP initiator of ε-caprolactone (CL) and δ-valerolactone (VL) were investigated by analysing aliquots of the reaction by ^1^H-NMR spectroscopy after set times (ESI Fig. 6, and 7†). From these samples, conversion of CL to poly(caprolactone) (PCL) and VL to poly(valerolactone) (PVL) could be calculated and plotted against time (Fig. 3). The conversion of CL and VL appear to follow first-order kinetics; with conversion of CL tending towards 100%, and conversion of VL approaching 85–90% after 5 hours (measured conversions are tabulated in ESI Tables 1 and 3†). The strain energy for δ-valerolactone is lower than that of ε-caprolactone.^37^ As a result of lower ring strain, polymerisation is less favourable for VL, leading to a lower conversion at equilibrium in comparison to CL.^37^ It is known in literature that VL exhibits a lower rate of polymerisation than CL in CaLB catalysed eROP.^36^ Targeting of different chain lengths by varying the initiator : monomer ratio (1 : 5, 1 : 10, 1 : 20, and 1 : 40) did not drastically alter the kinetics of monomer conversion. Similar conversions are obtained after 300 minutes of reaction time for all ratios of initiator : monomer studied.

**Fig. 4:**
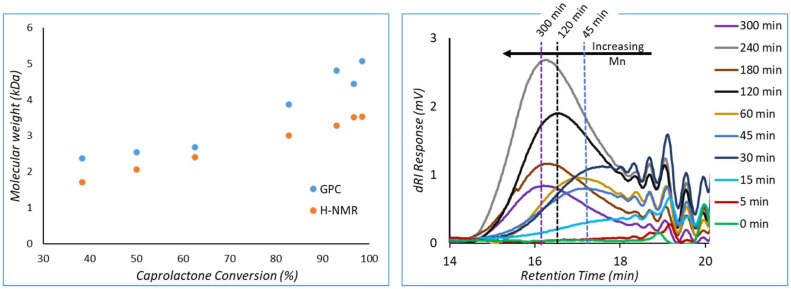
HEAA initiated eROP of CL (I : M 1 : 20) was monitored by both ^1^H-NMR analysis and GPC. Left: molecular mass (*M*_n_) determined by GPC and ^1^H-NMR spectroscopy plotted against conversion. Right: GPC chromatograms obtained during the experiment. Reactions were performed at a monomer concentration of 0.143 g mL^−1^.

HEAA initiated eROP of CL (1 : 20 I : M) was also monitored by GPC. From the chromatograms polymer growth and oligomeric materials can be observed. These oligomers were removed by precipitation into methanol (Fig. 8). However, unimodality is observed in the main polymer peak, suggesting a single species is forming. Molecular weight predicted by ^1^H-NMR and GPC are in good agreement, when partial conversion of HEAA is considered during molecular weight calculation (eqn (S5)†). When HEAA conversion is not considered molecular weight is significantly underestimated (ESI Table 2†). Discrepancies between molecular weight between GPC and ^1^H-NMR spectroscopy can arise from the GPC being calibrated to PMMA standards, which have different hydrodynamic volume compared to PCL.^38^ Furthermore, ^1^H-NMR does not have a lower limit of calibration, unlike GPC, resulting in oligomers being considered into the estimated molecular weight. Oligomers will be below the lower limit of calibration by GPC (around 800 Da), leading to overestimation of molecular weight in comparison to ^1^H-NMR (Fig. 4, left). Dispersity increases over time (ESI Table 2†), potentially due to transesterification taking place during polymerisation, although remains reasonably low (*Đ* = 1.28 at 30 min, to *Đ* = 1.51 at 300 min) (Fig. 4, right).

MALDI-TOF mass spectroscopy was performed on a sample of HEAA-PCL after purification by precipitation (ESI Fig. 10†). The obtained mass spectrum shows species with Na^+^ and K^+^ counterions (*m*/*z* difference of 16) but very clearly there is just a single polymer species observed. Difference in *m*/*z* of 114 between each additional unit correlating excellently with addition of single monomer units of caprolactone. Unfortunately, end-groups could not be accurately determined from MALDI alone. Mass determined by ^1^H-NMR end-group analysis (1797 g mol^−1^) and by MALDI (most intense peak 2104.5 *m*/*z*) are in close agreement, indicating that the primary species present is HEAA initiated PCL. However, as seen from the control experiments performed, residual water bound to N435 is also capable of acting as an initiator. Therefore, the presence of water-initiated chains cannot be excluded and is a limitation of the reaction methodology.

Turnover frequency for these reactions was calculated (ESI Table 4†) using literature methodology.^39^ Turnover frequencies are significantly higher in the initial stages of reaction. From the recyclability study (see ESI†) it can be ascertained that this decrease in turnover frequency is not a result of the enzyme losing catalytic activity. The decrease in turnover frequency is instead an effect of monomer concentration decreasing during reaction. An enzyme recyclability study was also performed (see ESI†). No loss in reactivity was observed over ten reaction cycles, demonstrating the robustness of the catalyst in the reaction conditions employed. Lactone and initiator conversion were consistent throughout the reaction cycles.

Control experiments without enzyme (reaction vessel containing HEAA and CL) and without HEAA (reaction vessel containing CL and N435) as initiator were also performed. No polymerisation of CL into PCL was observed upon removal of N435 (ESI Table 11†). On the other hand, upon removal of HEAA, polymerisation was observed between CL and N435. The polymerisation onset is likely due to residual water bound to the enzyme, as reported by previous literature.^40,41^ This background polymerisation proceeds significantly slower than when the HEAA initiator is added, and results in polymers of significantly higher dispersity. By GPC, polymer could only be detected after 2 hours of reaction, whereas when HEAA is added significant yield is seen within 30 minutes (ESI Fig. 11, and Table 5†). Whilst secondary initiation processes could not be removed entirely, the increased rate of reaction and the decreased dispersity can certainly be attributed to the presence of the desired initiator.

**Fig. 5:**
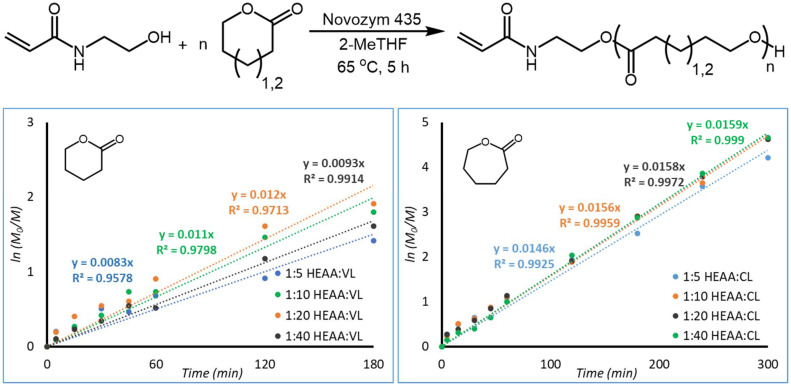
First-order plots of PVL (left) and PCL (right) formation. *M*_0_ and *M* were calculated using HEAA as an internal ^1^H-NMR standard. The last two data points for PVL were excluded. Reactions were performed at a monomer concentration of 0.143 g mL^−1^.

The natural logarithm of PCL and PVL formation were plotted against time to obtain a linear trend (Fig. 5). HEAA was used as an internal ^1^H-NMR standard to determine relative polymer concentration at sampling times, a more in-depth explanation and tabulated results can be found in the ESI† under the subsection “Plotting First-Order Kinetics”. Rate of reaction was found to be proportional to the concentration of lactone present, following first-order kinetics. VL does not appear to follow first order kinetics as closely as CL. For VL, the data points corresponding to 240 and 300 minutes were excluded, as the kinetics deviate from first-order kinetics as the reaction reaches equilibrium. *R*^2^ values exceed 0.95 when considering the first 180 minutes of reaction, indicating an adherence to first order kinetics for the initial stages of reaction. From conversion of the lactones into their respective poly(lactone)s it appears that this reaction proceeds following first order kinetics with respect to the lactone monomer. A deeper kinetic analysis contained in the ESI† enabled us to clarify in more detail the reactivity of VL and CL. VL was found to have significantly lower kinetic constants than CL, and to deviate from first-order kinetics (more details can be found in the section “Deeper Kinetics Analysis” in the ESI†).

#### Initiator consumption

The number of HEAA vinyl peaks was found to double in the ^1^H-NMR spectra during the kinetics studies of eROP (ESI Fig. 8†). Already after 30 minutes of reaction, formation of a new second species is apparent in the HEAA initiated eROP of CL and VL (ESI Fig. 6 and 7†). The observed increase in number of signals is believed to be a result of the presence of both unreacted HEAA and HEAA that has initiated a chain of PCL or PVL.

Purification was achieved by precipitation into methanol or diethyl ether, confirming that the increase in ^1^H-NMR signals is due to unreacted initiator rather than initiator degradation or self-reaction (ESI Fig. 9†). No extra peaks related to side products (*i.e.* ethanolamine, ESI Fig. 4†) were detected. In contrast, HEMA initiated eROP suffers from the targeted product and side products having almost identical solubility characteristics, and as a result purification by precipitation is impossible (ESI Fig. 1†).^5^

**Fig. 6:**
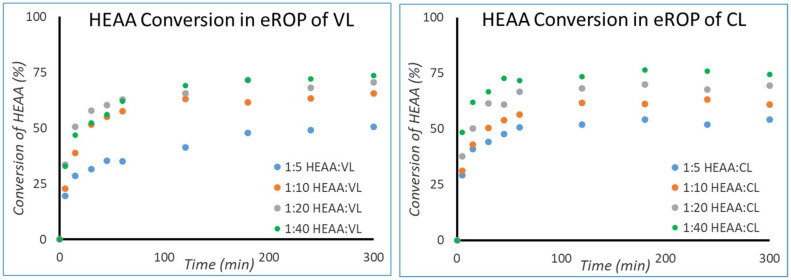
Kinetics of initiator conversion for HEAA initiated eROP of VL (left) and CL (right) in 2-MeTHF with 10 wt% N435. Note that HEAA is not fully consumed, unlike the lactone monomers, meaning that there is still unreacted HEAA present at the end of the reaction when all lactone has been consumed. Reactions were performed at a monomer concentration of 0.143 g mL^−1^.

It was possible to calculate the extent of initiator conversion using the two double doublets between 6.31 and 6.25 ppm corresponding to protons A and 1 (ESI Fig. 6 and 7†). Initiator consumption *versus* time is plotted in Fig. 6.

HEAA reaches conversions significantly lower than the conversions observed for CL and VL. As a result, targeted DP is exceeded, and unreacted HEAA can be found in the reaction mixture. As longer chains are targeted greater initiator conversion is observed. This is thought to be an effect of lowered reactivity as the poly(lactone) chain grows, due to increased steric hindrance.

It was hypothesised that chains of HEAA-PCL and HEAA-PVL are better substrates for N435 than HEAA, and therefore react faster than HEAA. The higher rate of monomer consumption by growing chains would in turn lead to complete monomer conversion before complete initiator conversion has been achieved. To further understand this phenomenon, HEAA and HEAA-CL were docked into the N435 active site using computational methods.

### Computational modelling of enzyme–substrate interaction

**Fig. 7:**
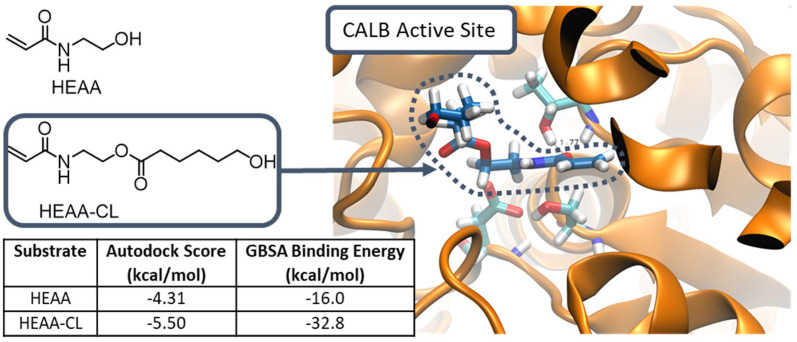
HEAA-CL docked in the active site of CALB (right), Autodock scores (kcal mol^−1^) and GBSA computed interaction energy (kcal mol^−1^) for HEAA and HEAA-CL (bottom left). A lower score indicates a higher binding affinity.

Computational studies were carried out to assess the interactions of HEAA and HEAA-PCL in the active site of CALB. Compounds were docked into the active site of N435 and then 10 ns molecular dynamics simulations were performed, with the interaction energies obtained using the Generalized Born Surface Area (GBSA) method within the Amber software package (Fig. 7).

From GBSA computed interaction energies, HEAA-CL binds approximately twice as strongly as HEAA. Based on the Autodock scores the difference is less pronounced, but HEAA-CL still exhibits a greater binding affinity to CALB than HEAA. Autodock scores and GBSA energies are obtained through different methods. Although the absolute values are not directly comparable, the trends between the compounds are of interest to gauge relative binding affinity to the enzyme active site.

To grow a poly(lactone) chain using N435, the activated monomer unit needs to be released from the acyl-enzyme intermediate in the rate-determining step. The release of monomer and regeneration of the enzyme occurs through nucleophilic attack by either an initiator or a propagating chain (ESI Fig. 17†).^36^ The rate of reaction is highly dependent on the binding interactions between enzyme and substrate.^42^ The higher affinity of HEAA-CL towards CaLB compared to HEAA rationalises the experimental results observed during eROP. Since HEAA-CL has a higher binding affinity than HEAA, it is more likely to be found in the enzyme active site and will subsequently react at a faster rate than HEAA. As a result, reactions involving propagating chain proceed at a faster rate than initiation, yielding unreacted initiator when all monomer has been consumed.

To obtain complete initiator conversion, the initiator should react at a faster rate than the propagating chains. To achieve rapid initiation an alternative initiator species that is more reactive in the presence of N435 could be used (HEMAM was screened, see “Alternative Initiator Species” in the ESI†), alternatively the enzyme active site could be modified to improve the suitability of HEAA as substrate although this is beyond the scope of the current work.

### Post-eROP reactions

To evaluate the quality of the produced degradable hybrid macromonomers two set of experiments were conducted. Organocatalyzed ROP of lactide has previously been used to demonstrate presence of a hydroxyl end group on the produce ROP product.^4^ FRP was used as a proof of concept to showcase the ability of the acrylamide end-group to partake in an industrially relevant reaction and to incorporate hydrophilicity *via* co-polymerisation. Reactivity observed at either end of HEAA initiated ROP products further substantiates the controlled nature of these reactions and products obtained.

### Chain extension using lactide

**Fig. 8:**
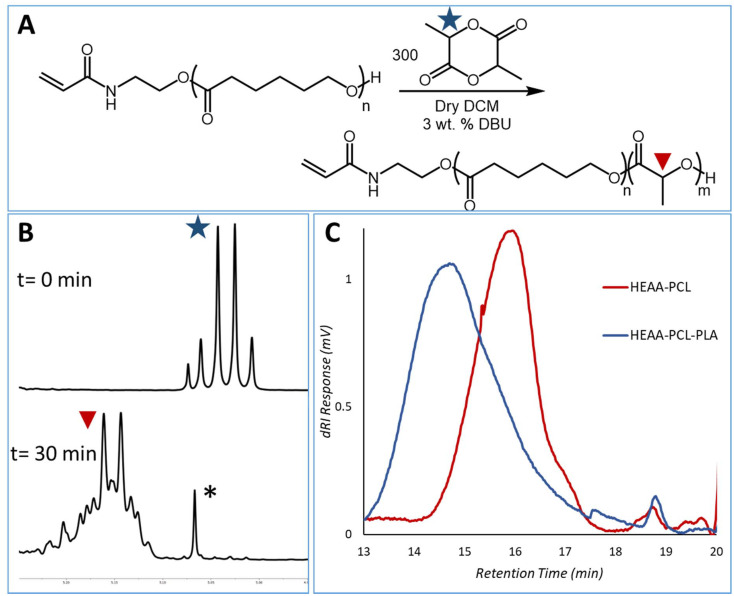
(A) Reaction scheme of HEAA-PCL extension with lactide using DBU. (B) ^1^H-NMR spectra of time zero and after 30 minutes, zoomed to 5.05–5.25 ppm – full spectra in ESI Fig. 18.† Peak marked with * is a DCM satellite. (C) GPC traces of HEAA-PCL (GPC *M*_n_ 9.45 kDa, *Đ* 1.2, ^1^H-NMR 4.5 kDa) and HEAA-PCL-PLA (GPC *M*_n_ 19.4 kDa, *Đ* 1.2, ^1^H-NMR 43 kDa) demonstrating a single polymer peak in both chromatograms.

Hydroxyl end-group fidelity of produced HEAA-PCL was demonstrated by chain extension using organocatalyzed ROP of lactide.^4^ The use of two distinct catalytic systems also showcases a methodical approach to overcome the limitations of DBU towards lactones and N435 towards lactide. 1,8-Diazabicyclo[5.4.0]undec-7-ene (DBU) has been demonstrated as an excellent organocatalyst for the ROP of lactide using labile-ester containing functional initiators (*i.e.* HEMA, PEGMA).^33^ Spurred on by the compatibility of DBU with labile-ester type initiators, it was expected this catalyst would not pose any issues in the presence of the less reactive amide containing HEAA. A purified sample of HEAA-PCL was used for the chain extension to prevent presence of HEAA in the sample. Successful chain extension was demonstrated by ^1^H-NMR spectroscopy and GPC analysis (Fig. 8).

In order to produce an A–B (A: PCL, B: PLA) block copolymer, we exploited the stability of the first block (HEAA-PCL) polymer and the compatibility of DBU with HEAA and 2-MeTHF. Lactide was added in a ratio of 300 : 1 with respect to HEAA-PCL and allowed to react for 30 minutes. The organocatalyzed ROP was monitored by ^1^H-NMR spectroscopy. Ring-opening of lactide is monitored using the peaks at 5.05 (quartet) and 5.25 ppm (broad multiplet) corresponding to monomeric lactide and polymeric PDLLA respectively. Conversion was found to be approximately 98% by ^1^H-NMR analysis. The successful chain extension and subsequent formation of a blocky system was confirmed by the presence of one single peak in the GPC chromatogram (with increased final number average molecular mass). Furthermore, chain extension of PCL with lactide leads to opportunities for tuneable biodegradability to the materials.

### Synthesis of hybrid polymers in 2-MeTHF

**Fig. 9:**
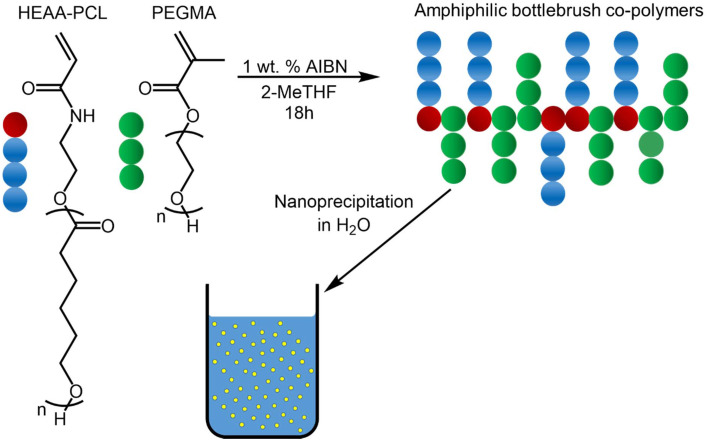
Synthetic strategy for the co-polymerisation of HEAA-PCL with PEGMA and subsequent nanoprecipitation into water.

Combining polymerisation techniques such as FRP and ROP facilitates the synthesis of amphiphilic hybrid polymers capable of self-assembly into biodegradable NPs targeting application in biomedicine.^12,13,43^ Our chosen strategy utilizes the synthesis of radically polymerisable macromonomers by initiating eROP using HEAA as a functional initiator. Labile-ester initiators HEMA and PEGMA are well known in this field. However, as discussed previously, they present issues of transesterification.^1,6^ FRP model reactions yielded amphiphilic grafted biodegradable co-polymers. Hybrid materials can potentially possess tuned degradation profiles and limited toxicity relative to simple FRP products (Fig. 9).^12^

PEGMA was chosen as the hydrophilic co-monomer due to its excellent biocompatibility,^44,45^ and widespread use in NP formation for drug delivery applications.^46,47^ Presence of a hydrophilic moiety such as PEGMA stabilized the NPs formed in aqueous media. FRP of a non-precipitated sample of HEAA-PCL (*i.e.* also containing unreacted HEAA due to incomplete initiator consumption observed) demonstrated an enhanced ability to assemble into NPs thanks to the hydrophilic HEAA present. Suitability of HEAA as a hydrophilic co-monomer removes the need for precipitation after eROP, improving the green credentials of the reaction by limiting purification steps and solvent requirements.

Significant discrepancies between molecular weight values (expected *versus* those measured by GPC) were observed for the FRP products. These effects are likely due to significant chemical differences between synthesised amphiphilic materials and the PMMA standards used for calibration. This has been previously described in literature^38^ and might be a result of different solvated volumes and column-polymer interactions (Table 2).

**Table 2:** Data for FRP (co-)polymerisations using HEAA-PCL macromonomers

Polymer	Ratio (HEAA-PCL) : comonomer	Conversion^a^ (%)	*M*_n_ GPC^b^ (kDa)	*M*_w_ GPC^b^ (kDa)	*Đ* b
Poly-(HEAA-PCL)_44_	1 : 0	Quant.	14.1	21.9	1.6
(HEAA-PCL)_44_-*co*-PEGMA	4 : 6	99	5.39	9.66	1.8
(HEAA-PCL)_44_-*co*-PEGMA	7 : 3	94	11.2	15.8	1.4
Poly-(HEAA-PCL)_15_	1 : 0	Quant.	9.18	13.2	1.4
(HEAA-PCL)_15_-*co*-PEGMA	4 : 6	98	4.00	5.36	1.3
(HEAA-PCL)_15_-*co*-PEGMA	7 : 3	99	5.71	8.87	1.6
(HEAA-PCL)_10_-*co*-HEAA	Approx. 1.80 : 0.17	Quant.	8.90	15.4	1.7

a Determined by ^1^H-NMR spectroscopy deemed quantitative when no olefinic protons visible in the spectrum.

b Determined by GPC in THF.

### Nanoparticle formation

The produced materials (purified macromonomers, FRP homopolymers, and FRP copolymers with PEGMA) were tested for their ability to self-assemble into nanoparticles upon nanoprecipitation into water. Presence of a hydrophilic moiety was found to significantly improve the ability of the materials to form nanoparticles. Macromonomers and FRP homopolymers, being almost entirely hydrophobic, failed to self-assemble and yielded only poorly defined aggregates. FRP copolymers of (HEAA-PCL)-*co*-PEGMA and (HEAA-PCL)-*co*-HEAA, aided by hydrophilic contributions from PEGMA and HEAA respectively, were found to self-assemble (Table 3). From the DLS plots (ESI Fig. 28†) it can be observed not all distributions are perfectly unimodal. This is a result of the samples not being filtered before measurement, leading to some aggregation being observed.

**Table 3:** DLS Measurements of formed nanoparticle suspensions

Entry	Polymer	Ratio (HEAA-PCL) : comonomer (mass : mass)	Hydrodynamic diameter (nm)	PDI
1	(HEAA-PCL)_44_-*co*-PEGMA	4 : 6	104.3 ± 1.2	0.189 ± 0.005
2	(HEAA-PCL)_44_-*co*-PEGMA	7 : 3	107.6 ± 1.1	0.213 ± 0.001
3	(HEAA-PCL)_15_-*co*-PEGMA	4 : 6	120.2 ± 4.4	0.556 ± 0.039
4	(HEAA-PCL)_15_-*co*-PEGMA	7 : 3	141.1 ± 0.8	0.483 ± 0.004
5	(HEAA-PCL)_10_-*co*-HEAA	Approx. 1.80 : 0.17	310.8 ± 3.5	0.235 ± 0.005

### Cytotoxicity

**Fig. 10:**
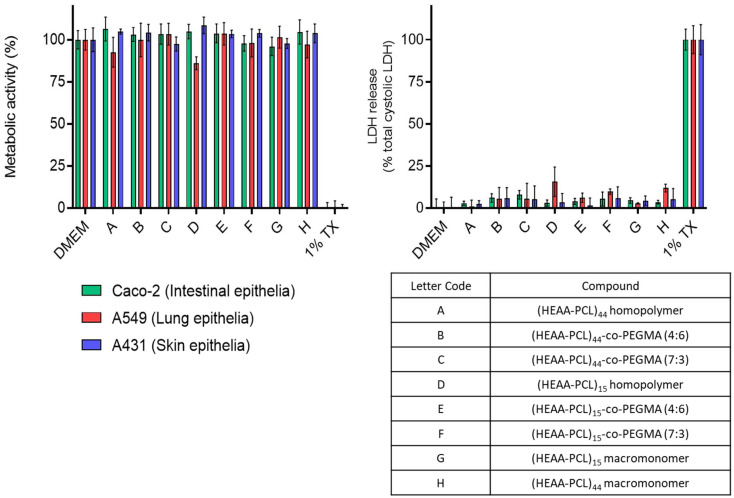
Biocompatibility of compounds on Caco-2 (intestinal), A549 (airway) and A431 (skin) epithelial cells. Cytotoxicity was determined by PrestoBlue metabolic activity and LDH release as an indicator of membrane damage. Compounds (500 μg mL^−1^) were applied to cells in 10% FBS containing DMEM and exposed for 48 hours to cells. DMEM treatment represents the vehicle control and Triton X-100 (TX) applied at 1% (v/v) was used as the cell death control. Data are presented as mean ± S.D.

Considering these materials might have potential application as biomaterials, their cytotoxicity was tested against several *in vitro* human cell line models. Intestinal epithelial (Caco-2), lung epithelial (A549), and skin epithelial (A431) cell lines were chosen to model oral, inhalation, and topical exposure routes, respectively. From Fig. 10, it can be seen that metabolic activity is maintained for the cell lines exposed to the synthesised polymeric materials and DMEM (vehicle control). LDH release was assayed as an indication of damage to the cell membranes. In the cases of DMEM and polymeric materials this release was minimal, indicating an absence of cell membrane damage. Together the data demonstrate that the materials exhibit minimal cytotoxicity at concentrations up to 500 μg mL^−1^.

## Conclusions

In this work we demonstrate the use of HEAA as a functional initiator for eROP using Novozym 435 as a green catalyst in a bio-renewable solvent (2-MeTHF). The HEAA amide bond is stable towards N435, enabling the synthesis of biodegradable aliphatic polyesters with a functional handle using enzymatic catalysis, and overcoming the initiator degradation *via* transesterification that is observed in HEMA initiated eROP. From kinetics studies it was found that caprolactone follows first-order kinetics, whereas valerolactone deviates from ideal first-order behaviour. Incomplete initiator conversion was observed, meaning not all of the HEAA present during reaction was able to initiate a polymer chain. Using computational docking techniques, we have determined that the incomplete initiator consumption can be attributed to initiated polymer chains having a higher affinity for the active site on N435. As a result, initiation was outcompeted by propagation, and resulted in leftover initiator when all monomer has been consumed. By minimising side reactions only mono-functional hybrid macromonomers have been prepared for the first time *via* an eROP approach.

HEAA-PCL was carried forward to produce amphiphilic graft copolymers. As a proof of concept of a simple and largely adopted class of reaction, FRP with PEGMA as a co-monomer was performed. This FRP co-polymerisation yielded materials that were capable of self-assembly into NPs, whereas purely hydrophobic homopolymers only yielded poorly defined aggregates. One-pot eROP and subsequent FRP of crude HEAA-PCL, still containing unreacted and hydrophilic HEAA, also yielded material capable of self-assembly into NPs. The ability of unreacted HEAA to aid in NP formation can be exploited to carry out eROP and subsequent FRP in one-pot, minimizing synthetic steps and solvent use. Materials were assayed for cytotoxicity against three model cell lines (Caco-2, A549, and A431) to determine their viability for application in biological context, and found to be non-toxic at concentrations up to 500 μg mL^−1^.

## Experimental section

### Materials

Novozym 435 lipase ([9001-62-1], derived from *C. antarctica* (>5000 U g^−1^) and immobilized on an acrylic macroporous resin, was kindly donated by Novozymes A/S, Denmark. ε-Caprolactone, δ-valerolactone, PEGMA (300 Da average *M*_n_) were purchased from Fischer Scientific and stored over molecular sieves. HEAA, and 2-MeTHF, were purchased from Sigma Aldrich and used as received. THF and methanol were purchased from Fischer Scientific UK and used without further purification unless otherwise stated. Water was deionised before use.

### General methods and instrumentation

#### Nuclear magnetic resonance spectroscopy (NMR)

Polymer formation and chemical structure assignment was determined using ^1^H-NMR spectroscopy. Approximately 4 mg of sample was dissolved in 0.7 mL of DMSO-*d*_6_ or CDCl_3_ and analysed using a Bruker DPX 400 MHz spectrometer operating at 400 MHz (^1^H), assigning chemical shifts in parts per million (ppm). MestReNova 6.0.2 copyright 2009 (Mestrelab Research S.L.) was used for analysing the spectra.

#### Gel permeation chromatography (GPC)

was performed in THF (HPLC grade, Fisher Scientific) as the eluent at 40 °C using two Agilent PL-gel mixed-D columns in series, an injection loop of 50 μL, with a flow rate of 1 mL min^−1^. A differential refractometer (DRI) was used for the detection of samples. Samples were made at a concentration of approximately 2 mg mL^−1^ in HPLC grade THF, and filtered through a 0.22 μm Teflon filter before injection. The system was calibrated using poly(methyl methacrylate) standards with average molecular weight in the range from 540 to 1.02 × 10^6^ g mol^−1^ and dispersity (*Đ*) close to 1.0.

#### Dynamic light scattering (DLS)

Particle size analyses were performed by DLS utilizing a Zetasizer Nano spectrometer (Malvern Instruments Ltd) equipped with a 633 nm laser at a fixed angle of 173°. Nanoparticles were prepared at a concentration of 1 mg mL^−1^ adopting a simple solvent displacement methodology (acetone/DI H_2_O ratio 1 : 5). Samples were equilibrated at 25 °C for 30 seconds prior to measurements. All experiments were performed in duplicate averaging 10 scans per run of the same sample.

#### Matrix assisted laser desorption ionisation – time of flight mass spectroscopy (MALDI-TOF MS)

Polymer formation was assessed on a Bruker Ultraflex III mass spectrometer. *trans*-2-[3-(4-*tert*-Butylphenyl)-2-methyl-2-propenylidene]malononitrile (DCTB) was used as the matrix. Matrix (20 μL, 20 mg mL^−1^ in acetonitrile) and polymer (10 μL, 10 mg mL^−1^ in acetonitrile) were mixed, and the mixture (0.5 μL) was spotted onto the MALDI sample plate. PEG was used for calibration. Spectra were recorded at 50% laser power.

#### HEAA initiated eROP of CL or VL and recyclability study

In a typical procedure CL (1.00 g, 8.76 mmol), HEAA (0.2 to 0.025 mol equiv.), and solvent (2-MeTHF, 7 mL, monomer concentration of 0.143 g mL^−1^) were combined and stirred until homogeneous. Novozym 435 (10 wt%, 0.1 g) was added, and the reaction was stirred and heated (100 rpm, 65 °C). The mixture was filtered to remove the catalyst, and solvent was removed by rotary evaporation to collect the crude polymeric product. Alternatively, the reaction mixture was precipitated thrice into a mixture of cold mixture of methanol and diethyl ether to obtain a polymeric sample free of unreacted initiator.

For the enzyme recyclability study, the same procedure as above was followed, where the Novozym 435 was collected and re-used.

For the control experiments, the same procedure as above was followed, excluding addition of initiator.

### Lactide extension of HEAA-PCL

A precipitated sample of HEAA-PCL (*M*_n_ 4500 g mol^−1^ by ^1^H-NMR analysis) (0.0401 g, 0.00891 mmol), and lactide (0.450 g, 3.12 mmol) were dissolved in dry DCM (6 mL). DBU (3 mol%) was subsequently added. Upon completion, monitored by ^1^H-NMR spectroscopy, the reaction was precipitated into an ice-cold mixture of diethyl ether and petroleum ether (70 : 30 v : v, 35 mL). The product was collected by centrifugation and allowed to dry *in vacuo*.

### Computational docking

Compounds were docked with Autodock version 4.2^48^ on the reported crystal structure of lipase B from *Candida antarctica* (PDB code: 1LBS). A docking grid with a spacing of 0.375 Å was generated. 500 genetic algorithm runs were performed and the binding mode with lowest energy was selected. Explicit ligand hydrogen atoms were used and Gasteiger charges were added by Autodock. Molecular dynamics (MD) simulations were carried out using the GPU accelerated version of Amber 20.^49^ Complexes were built starting from the docking conformation using the tleap module of AmberTools21.^49^ The Amber FF19SB force field^50^ was used for the protein and GAFF^51,52^ with BCC charges for the ligands. A sodium ion was added to neutralize the system and the complex was solvated in an octahedral box of TIP3P^53^ water molecules extending 10.0 Å from the protein. Periodic boundary conditions were applied. 1000 cycles of steepest descent minimization were followed by 1000 cycles of conjugate gradient minimization. The system was then heated to 300 K for 20 ps using Langevin dynamics with a collision frequency of 2.0 ps^−1^ with no constant pressure scaling, and was then equilibrated for 60 ps at 300 K at constant pressure with isotropic position scaling. The production run consisted of a 10 ns MD simulation at 300 K with no constant pressure scaling and SHAKE^54^ for bonds involving hydrogen. Binding energy calculations were performed with the MMPBSA.py package in AmberTools21. 5000 snapshots from the 10 ns MD simulation were evaluated using the Generalized Born solvent model II^55^ and a salt concentration of 0.1 M.

### FRP general procedure

Monomers were combined with 2-MeTHF (25 wt% monomer loading) and allowed to dissolve before cooling (0 °C), and addition of AIBN (1 wt% with respect to monomers). The reaction mixture was degassed with argon (0.5 h, 0 °C) before being heated (65 °C, 22 h). The polymerisations were halted by exposure to air and precipitation into an excess of ice-cold diethyl ether. The precipitates were cooled (−20 °C) before centrifugation (4500 rpm, 10 min), followed by decantation of the supernatant and drying of the resulting polymers.

### One-pot eROP and subsequent FRP

HEAA (0.336 g, 2.92 mmol), CL (1.66 g, 14.6 mmol), and 2-MeTHF (10 mL, 23 wt% monomer loading) were combined in a vial and stirred until homogenous. Novozym 435 (10 wt% wrt monomer, 0.2 g) was added, and the reaction mixture was heated (65 °C, 18 h). Novozym 435 was removed by filtration, the remaining solution was cooled (0 °C), AIBN (1 wt%, 20 mg) was added, and the mixture was degassed using argon (30 min) before sealing and heating (65 °C, 22 h). The polymerisations were halted by exposure to air and precipitation into an excess of ice-cold methanol (35 mL). The precipitates were cooled (−20 °C) before centrifugation (4500 rpm, 10 min), followed by decantation of the supernatant and drying of the resulting polymers.

### Nanoparticle formation

Nanoparticles were prepared by a nanoprecipitation method. Polymers (10 mg) were dissolved in acetone (1 mL). The polymeric solution was then added dropwise to deionised water (10 mL) under constant stirring at 550 rpm. Nanoparticle dispersions were formed through solvent exchange between water and acetone. The final dispersion was then left stirring overnight at room temperature in order to reach complete acetone evaporation, final NPs concentration of 1 mg mL^−1^. The water level was marked before addition of the polymer solution, and complete solvent evaporation was assumed when the level had returned to the same mark.

### Cytotoxicity evaluation

Caco-2 human colonic cancer epithelial, A549 human lung adenocarcinoma and human epidermoid carcinoma cells were obtained from the American Type Culture Collection (ATCC; Manassas, Virginia) and used at passages 35–40, 30–35 and 25–30, respectively. Cells were cultured in DMEM (Sigma-Aldrich) supplemented with 10% (v/v) FBS (Sigma-Aldrich) and 2 mM l-glutamine (Sigma-Aldrich), and at 37 °C with 5% CO2.

The lactate dehydrogenase (LDH) release assay (Sigma Aldrich, TOX7 kit) and PrestoBlue cell viability assay (Thermo Fisher Scientific) were performed to assess cytotoxicity. Cells were seeded at 1.2 × 10^5^ cells per well in 12 well plates and cultured for 24 hours prior to assaying. Polymeric materials were exposed to cells at 500 μg mL^−1^ for 48 hours and applied in 1 mL phenol red free DMEM containing 10% (v/v) FBS and 2 mM l-glutamine. Triton X-100 (TX) applied at 1% (v/v) applied in phenol red free medium was used as a cell death (positive) control and a vehicle control containing no polymeric material used as a negative control. Following exposure, sample supernatant was collected from wells for analysis of LDH content. Cells were then washed twice with warm PBS and 10% (v/v) PrestoBlue reagent diluted in phenol red free medium applied per well for 60 minutes. The resulting fluorescence was measured at 560/600 nm (*λ*_ex_/*λ*_em_). Relative metabolic activity was calculated by setting values from the negative control as 100% and positive control values as 0% metabolic activity. Assessment of LDH release was performed according to the manufacturer's instructions and involved adding LDH reagent to collected supernatant samples and incubating at room temperature shielded from light for 25 minutes. Absorbance was then measured at 492 nm. Relative LDH release was calculated with the negative control absorbance at 492 nm taken as 0%, and the positive control, assumed to cause total cell lysis, as 100%.

## Conflicts of interest

There are no conflicts to declare.

## Supplementary Material

PY-013-D2PY00849A-s001

## References

[cit1] Ruiz-Cantu L. A., Pearce A. K., Burroughs L., Bennett T. M., Vasey C. E., Wildman R., Irvine D. J., Alexander C., Taresco V. (2019). Macromol. Chem. Phys..

[cit2] Ferrari R., Yu Y., Morbidelli M., Hutchinson R. A., Moscatelli D. (2011). Macromolecules.

[cit3] Kobayashi S. (2010). Proc. Jpn. Acad., Ser. B.

[cit4] Englezou G., Kortsen K., Pacheco A. A. C., Cavanagh R., Lentz J. C., Krumins E., Sanders-Velez C., Howdle S. M., Nedoma A. J., Taresco V. (2020). J. Polym. Sci..

[cit5] Takwa M., Xiao Y., Simpson N., Malmström E., Hult K., Koning C. E., Heise A., Martinelle M. (2008). Biomacromolecules.

[cit6] Xiao Y., Takwa M., Hult K., Koning C. E., Heise A., Martinelle M. (2009). Macromol. Biosci..

[cit7] Morales-Huerta J. C., Martínez De Ilarduya A., León S., Muñoz-Guerra S. (2018). Macromolecules.

[cit8] Goddard A. R., Apebende E. A., Lentz J. C., Carmichael K., Taresco V., Irvine D. J., Howdle S. M. (2021). Polym. Chem..

[cit9] Phan H., Kortsen K., Englezou G., Couturaud B., Nedoma A. J., Pearce A. K., Taresco V. (2020). J. Polym. Sci..

[cit10] Olsén P., Borke T., Odelius K., Albertsson A. C. (2013). Biomacromolecules.

[cit11] Zhao Y., He G., Guo W., Bao L., Yi M., Gong Y., Zhang S. (2016). Polym. Chem..

[cit12] Capasso Palmiero U., Sponchioni M., Manfredini N., Maraldi M., Moscatelli D. (2018). Polym. Chem..

[cit13] Guo X., Choi B., Feng A., Thang S. H. (2018). Macromol. Rapid Commun..

[cit14] Liang G., Wang A., Li L., Xu G., Yan N., Zhang T. (2017). Angew. Chem., Int. Ed..

[cit15] Kobayashi S., Makino A. (2009). Chem. Rev..

[cit16] Xu J., Song J., Pispas S., Zhang G. (2014). Polym. Chem..

[cit17] Tanzi M. C., Verderio P., Lampugnani M. G., Resnati M., Dejana E., Sturani E. (1994). J. Mater. Sci. Mater. Med..

[cit18] Li C., Pan S., Xu W., Lu Y., Wang P., Zhang F., Gross R. A. (2020). Green Chem..

[cit19] Ortiz C., Ferreira M. L., Barbosa O., dos Santos J. C. S., Rodrigues R. C., Berenguer-Murcia Á., Briand L. E., Fernandez-Lafuente R. (2019). Catal. Sci. Technol..

[cit20] Yamada R., Suzuki Y., Yasuda M., Ogino H. (2016). J. Supercrit. Fluids.

[cit21] Pellis A., Weinberger S., Gigli M., Guebitz G. M., Farmer T. J. (2020). Eur. Polym. J..

[cit22] Kortsen K., Pacheco A. A. C., Lentz J. C., Taresco V., Howdle S. M. (2021). J. Supercrit. Fluids.

[cit23] Narumi A., Chen Y., Soné M., Fuchise K., Sakai R., Satoh T., Duan Q., Kawaguchi S., Kakuchi T. (2009). Macromol. Chem. Phys..

[cit24] Sun Y., Fu L., Olszewski M., Matyjaszewski K. (2019). Macromol. Rapid Commun..

[cit25] Albarghouthi M. N., Stein T. M., Barron A. E. (2003). Electrophoresis.

[cit26] Bal A., Özkahraman B., Özbaş Z. (2016). J. Appl. Polym. Sci..

[cit27] Zhao C., Chen Q., Patel K., Li L., Li X., Wang Q., Zhang G., Zheng J. (2012). Soft Matter.

[cit28] Gong C. Y., Shi S., Dong P. W., Kan B., Gou M. L., Wang X. H., Li X. Y., Luo F., Zhao X., Wei Y. Q., Qian Z. Y. (2009). Int. J. Pharm..

[cit29] Hossaini R., Chipperfield M. P., Montzka S. A., Leeson A. A., Dhomse S. S., Pyle J. A. (2017). Nat. Commun..

[cit30] Aycock D. F. (2007). Org. Process Res. Dev..

[cit31] Pace V., Hoyos P., Castoldi L., Domínguez de María P., Alcántara A. R. (2012). ChemSusChem.

[cit32] Pellis A., Byrne F. P., Sherwood J., Vastano M., Comerford J. W., Farmer T. J. (2019). Green Chem..

[cit33] Pearce A. K., Vasey C. E., Anane-Adjei A. B., Sodano F., Crucitti V. C., Irvine D. J., Howdle S. M., Alexander C., Taresco V. (2019). Macromol. Chem. Phys..

[cit34] Pace V., Holzer W., Olofsson B. (2014). Adv. Synth. Catal..

[cit35] Kaiser D., Bauer A., Lemmerer M., Maulide N. (2018). Chem. Soc. Rev..

[cit36] Shoda S., Uyama H., Kadokawa J., Kimura S., Kobayashi S. (2016). Chem. Rev..

[cit37] Saiyasombat W., Molloy R., Nicholson T., Johnson A., Ward I., Poshyachinda S. (1998). Polymer.

[cit38] Al-Natour M. A., Yousif M. D., Cavanagh R., Abouselo A., Apebende E. A., Ghaemmaghami A., Kim D.
H., Aylott J. W., Taresco V., Chauhan V. M., Alexander C. (2020). ACS Macro Lett..

[cit39] Luna C., Verdugo C., Sancho E. D., Luna D., Calero J., Posadillo A., Bautista F. M., Romero A. A. (2014). Bioresour. Bioprocess..

[cit40] Deng F., Gross R. A. (1999). Int. J. Biol. Macromol..

[cit41] Uyama H., Takeya K., Hoshi N., Kobayashi S. (1995). Macromolecules.

[cit42] Corici L., Pellis A., Ferrario V., Ebert C., Cantone S., Gardossi L. (2015). Adv. Synth. Catal..

[cit43] Tian H., Tang Z., Zhuang X., Chen X., Jing X. (2012). Prog. Polym. Sci..

[cit44] Mastrotto F., Salmaso S., Lee Y. L., Alexander C., Caliceti P., Mantovani G. (2013). Polym. Chem..

[cit45] Hussain H., Mya K. Y., He C. (2008). Langmuir.

[cit46] Jia X., Zhao X., Tian K., Zhou T., Li J., Zhang R., Liu P. (2016). Chem. Eng. J..

[cit47] Agostini A., Capasso Palmiero U., Barbieri S. D. A., Lupi M., Moscatelli D. (2018). Nanotechnology.

[cit48] Morris G. M., Ruth H., Lindstrom W., Sanner M. F., Belew R. K., Goodsell D. S., Olson A. J. (2009). J. Comput. Chem..

[cit49] Case D. A. , Aktulga H. M., Belfon K., Ben-Shalom I. Y., Brozell S. R., Cerutti D. S., Cheatham III T. E., Cisneros G. A., Cruzeiro V. W. D., Darden T. A., Duke R. E., Giambasu G., Gilson M. K., Gohlke H., Goetz A. W., Harris R., Izadi S., Izmailov S. A., Jin C., Kasavajhala K., Kaymak M. C., King E., Kovalenko A., Kurtzman T., Lee T. S., LeGrand S., Li P., Lin C., Liu J., Luchko T., Luo R., Machado M., Man V., Manathunga M., Merz K. M., Miao Y., Mikhailovskij O., Monard G., Nguyen H., O'Hearn K. A., Onufriey A., Pan F., Pantano S., Qi R., Rahnamoun A., Roe D. R., Roitberg A., Sagui C., Schott-Verdugo S., Shen J., Simmerling C. L., Skrynnikov N. R., Smith J., Swails J., Walker R. C., Wang J., Wei H., Wolf R. M., Wu X., Xue Y., York D. M., Zhao S. and Kollman P. A., Amber, 2021, Univ. California, San Fr.

[cit50] Tian C., Kasavajhala K., Belfon K. A. A., Raguette L., Huang H., Migues A. N., Bickel J., Wang Y., Pincay J., Wu Q., Simmerling C. (2020). J. Chem. Theory Comput..

[cit51] Wang J., Wang W., Kollman P. A., Case D. A. (2006). J. Mol. Graphics Modell..

[cit52] Wang J., Wolf R. M., Caldwell J. W., Kollman P. A., Case D. A. (2004). J. Comput. Chem..

[cit53] Jorgensen W. L., Chandrasekhar J., Madura J. D., Impey R. W., Klein M. L. (1983). J. Chem. Phys..

[cit54] Ryckaert J. P., Ciccotti G., Berendsen H. J. C. (1977). J. Comput. Phys..

[cit55] Onufriev A., Bashford D., Case D. A. (2004). Proteins: Struct., Funct., Genet..

