# Supplementary material for: N-Hydroxyethyl acrylamide as a functional eROP initiator for the preparation of nanoparticles under “greener” reaction conditions
Source: Polym Chem. 2022 Oct 10;13(42):6032–45. doi: 10.1039/d2py00849a (PMC9623607; doi:10.1039/d2py00849a)
Supplement: PY-013-D2PY00849A-s001 [file PY-013-D2PY00849A-s001.pdf]

Electronic Supplementary Material (ESI) for Polymer Chemistry.
This journal is © The Royal Society of Chemistry 2022

Supporting Information

N-Hydroxyethyl acrylamide as a functional eROP initiator for the preparation of nanoparticles in "greener" reaction conditions

Joachim C. Lentz,[a] Robert Cavanagh,[b] Cara Maloney,[b] Bruno Falcone Pin,[a] Kristoffer Kortsen,[a] Harriet R. Fowler,[a] Philippa L. Jacob,[a] Eduards Krumins,[a] Charlotte Clark,[a] Fabricio Machado,[a,d] Nicholas Breitkreuz,[a] Ben Cale,[c] Amy R. Goddard,[c] Jonathan D. Hirst,[a] Vincenzo Taresco,[*a] and Steven M. Howdle.[*a]

a. *School of Chemistry, University of Nottingham, University Park, NG7 2RD, Nottingham, United Kingdom*
b. *School of Pharmacy, University of Nottingham, University Park, NG7 2RD, Nottingham, United Kingdom*
c. *Croda Europe Limited, Cowick Hall, Snaith, DN14 9AA, Goole, United Kingdom*
d. *Institute of Chemistry, University of Brasília, Campus Universitário Darcy Ribeiro, 70910-900, Brasília, DF, Brazil*

Equations

$$Transesterification\ of\ HEMA\ (\%) = \frac{\int 6.11\ ppm}{\int 6.13 ppm + \int 6.11\ ppm} \times 100$$

Equation S1 Transesterification of HEMA (%) was calculated by analysis of the peaks at 6.13 and 6.11 ppm in the H-NMR spectra, where the peak at 6.11 ppm corresponds to the transesterified species and the peak at 6.13 ppm corresponds to unreacted HEMA.

$$Conversion\ of\ CL\ to\ PCL\ (\%) = \frac{\int 2.36\ to\ 2.26\ ppm}{\int 2.61\ ppm + \int 2.36\ to\ 2.26\ ppm} \times 100$$

Equation S2 Conversion CL to PCL (%) was calculated by analysis of the peaks at 2.61 and 2.36 to 2.26 ppm in the H-NMR spectra, where the peak at 2.61 ppm corresponds to the unreacted CL and the peak at 2.36 to 2.26 ppm corresponds to PCL.

$$Conversion\ of\ HEAA\ to\ HEAA - PCL\ (\%) = \frac{\int 6.29\ ppm}{\int 6.31\ ppm + \int 6.29\ ppm} \times 100$$

Equation S3 Conversion HEAA to HEAA-PCL (%) was calculated by analysis of the peaks at 6.31 and 6.29 ppm in the H-NMR spectra, where the peak at 6.31 ppm corresponds to unreacted HEAA and the peak at 6.29 ppm corresponds to reacted HEAA.

$$DP\ of\ (HEAA - PCL) = \frac{PCL\ units}{Reacted\ HEAA\ units} = \frac{CL_0 \times \frac{CL\ conversion}{100}}{\frac{HEAA\ conversion}{100}}$$

Equation S4 DP of HEAA-PCL can be estimated from the ratio of reacted monomer to the ratio of reacted initiator. Where CL_0 is the number of CL moles present at time zero divided by the number of HEAA moles present, i.e. 5, 10, 25.

$$Estimated\ MW\ of\ HEAA - PCL = MW\ of\ HEAA + (DP\ of\ HEAA - PCL\ \times MW\ of\ CL)$$

Equation S5 Estimation of MW from DP can be made assuming each chain is initiated by HEAA.

Stability Screening of HEAA towards N435

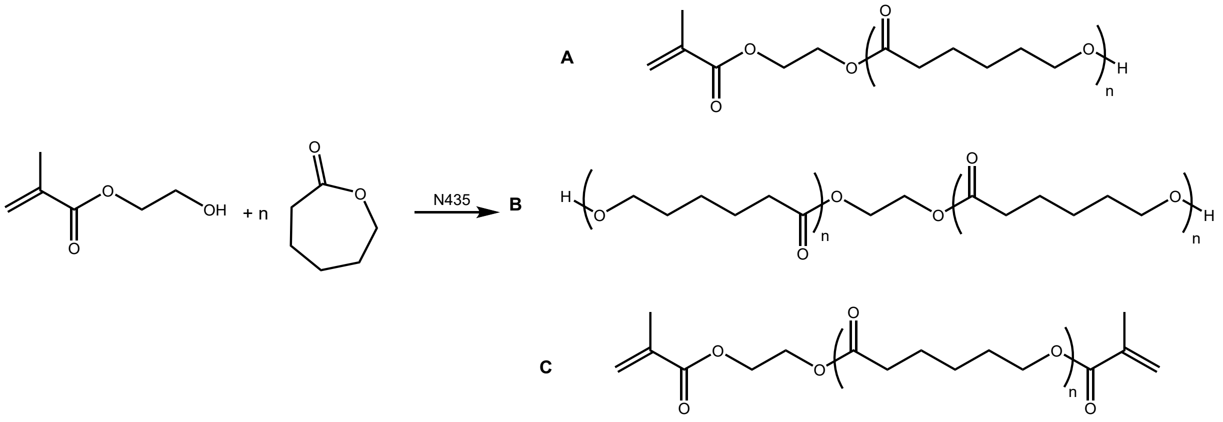

SI Figure 1 HEMA initiated eROP yields a mixture of products. A: Desired product, B: Ethylene glycol initiated PCL chains (unreactive in FRP), and C: PCL chains featuring two methacrylate end groups (cross-linkers in FRP).

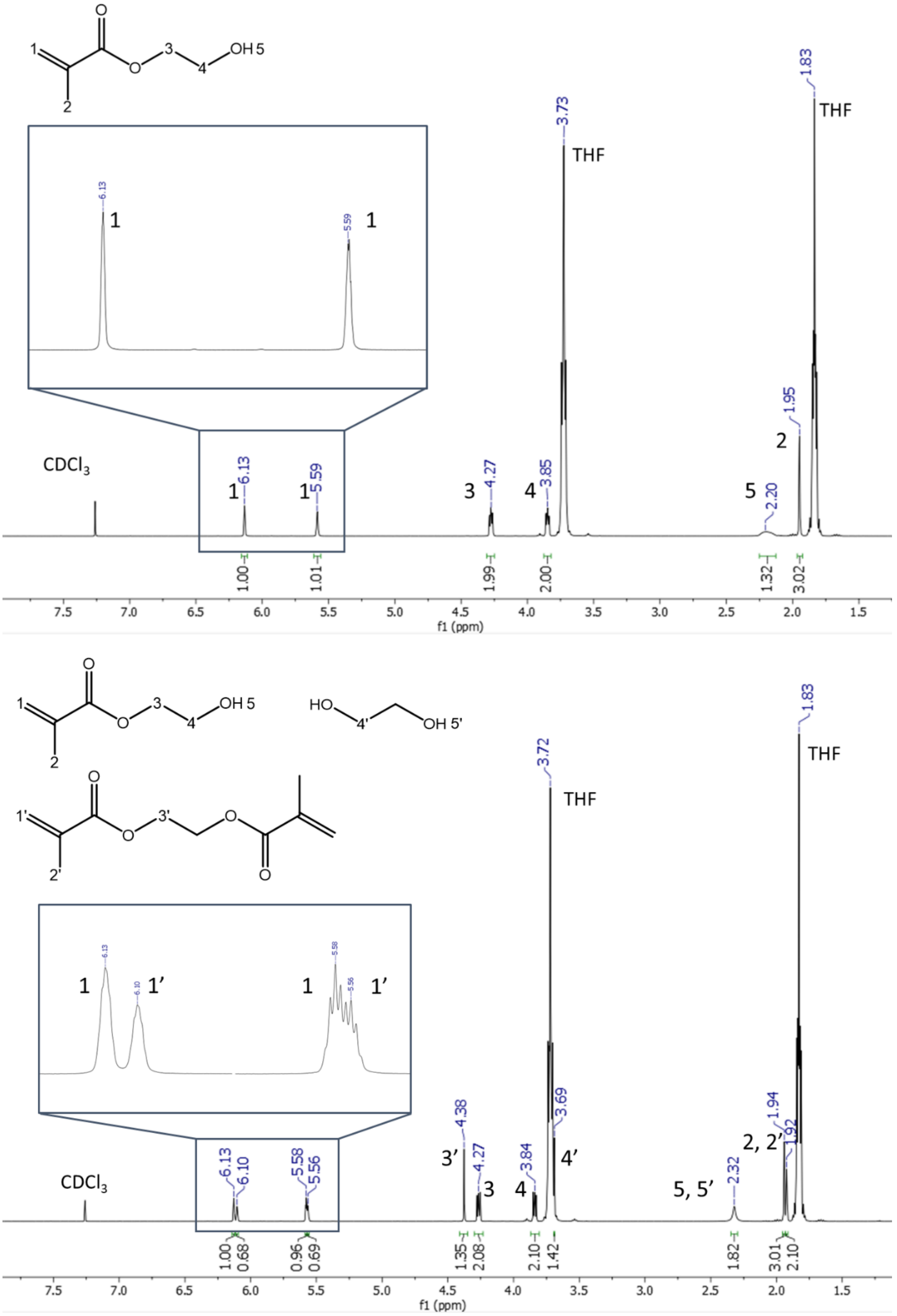

SI Figure 2 ^{1}H-NMR spectrum of HEMA in $CDCl_3$ before (top) and after 20 h at 65 °C with 20 wt. % N435 (bottom) with an indent to focus on the vinyl region. Note the appearance of new peaks corresponding to ethylene glycol dimethacrylate and ethylene glycol in the lower spectrum.

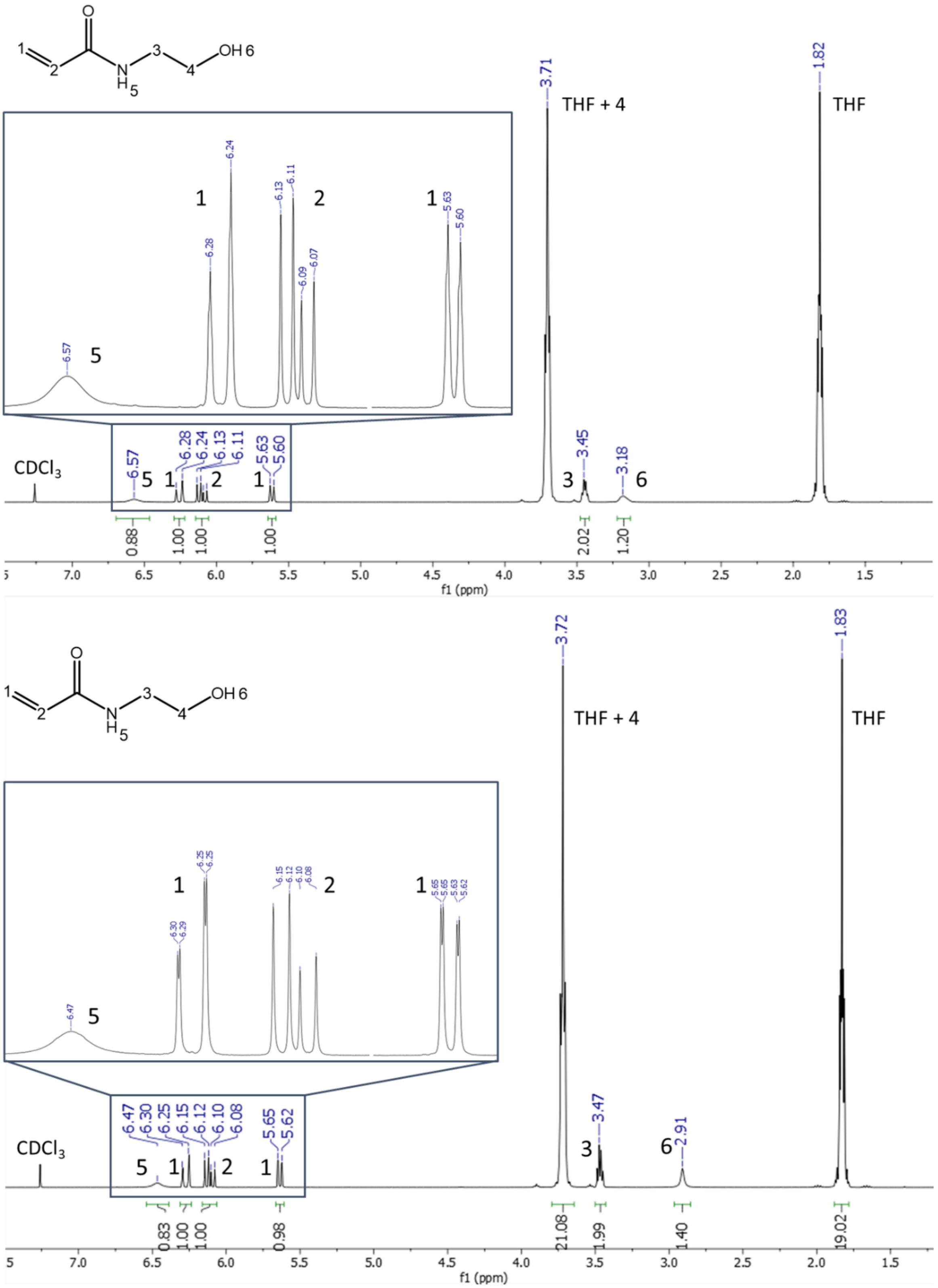

SI Figure 3 ^{1}H-NMR spectrum of HEAA and THF in $CDCl_3$ before (top) and after 20 h at 65 °C with 20 wt.% N435 (bottom). Note the lack of change before and after subjecting HEAA to reaction conditions, indicating that no reaction has taken place. In the lower spectrum the fine splitting in the peaks labelled 1 can be resolved to see a doublet of doublets. Note that the peak corresponding to the protons of 4 are hidden underneath a peak corresponding to THF.

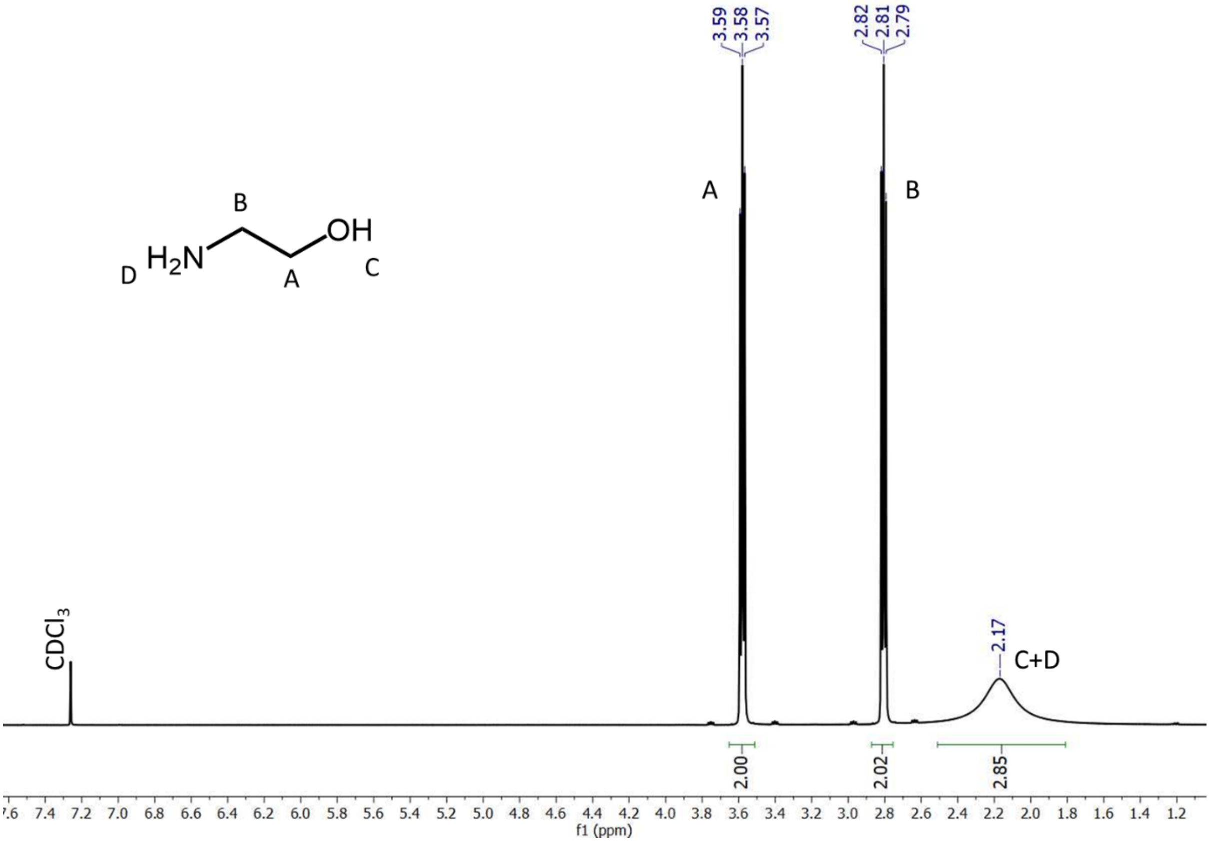

SI Figure 4 ^{1}H-NMR spectrum of ethanolamine in $CDCl_3$.

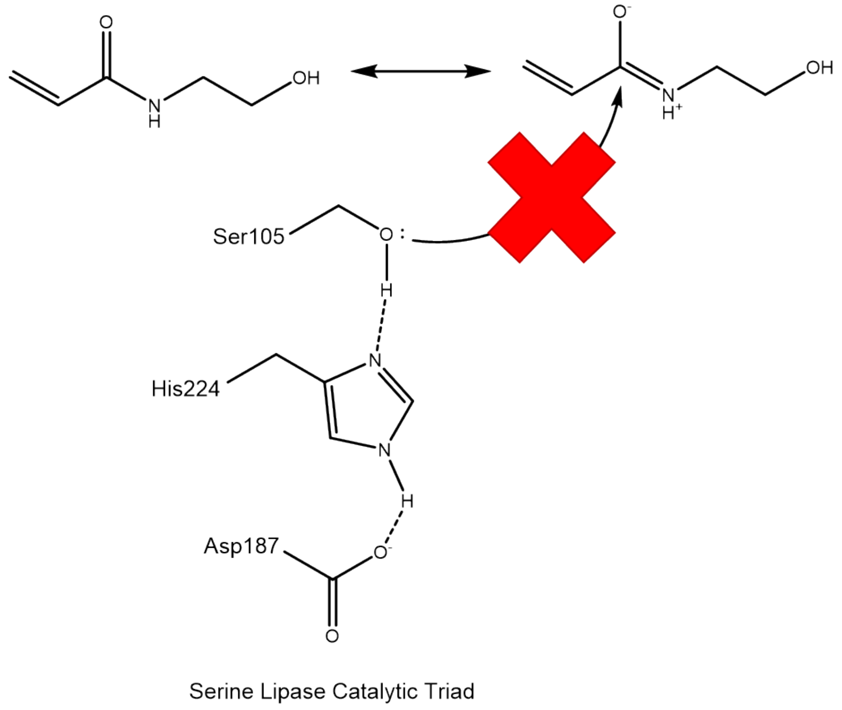

SI Figure 5 It was hypothesised that delocalisation of the nitrogen lone pair in HEAA acts to deactivate the carbonyl bond towards nucleophilic attack by the activated serine residue in CaLB.

Kinetics Data

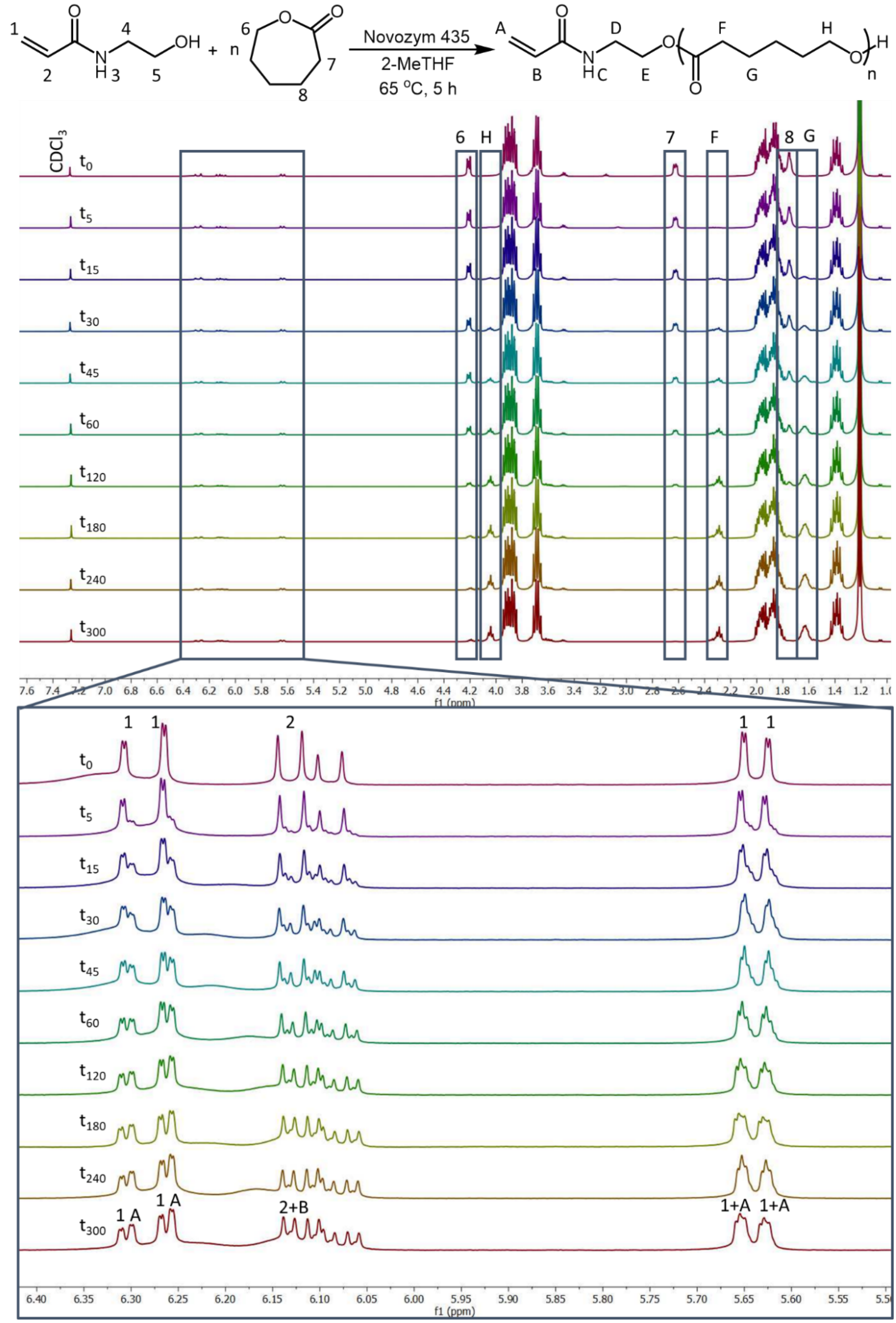

SI Figure 6 Full stack of ^{1}H-NMR spectra used to monitor the kinetics of HEAA initiated eROP of CL (M:I = 5:1). Zoomed in region highlights the formation of two distinct species of HEAA. Monomer concentration of 0.143 gmL^{-1}.

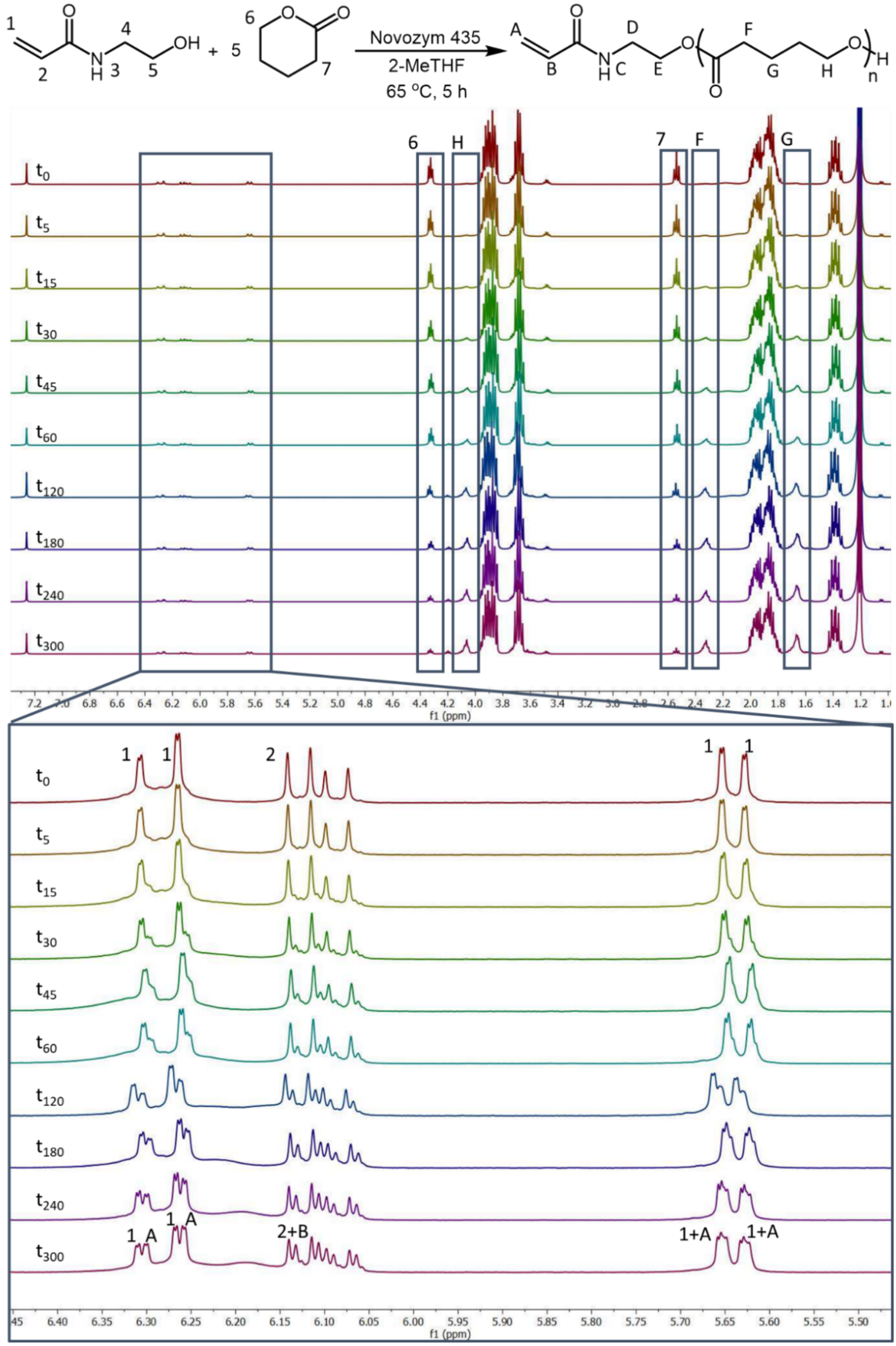

SI Figure 7 Full stack of ^{1}H-NMR spectra used to monitor the kinetics of HEAA initiated eROP of VL (M:I = 5:1). Zoomed in region highlights the formation of two distinct species of HEAA. Monomer concentration of 0.143 gmL^{-1}.

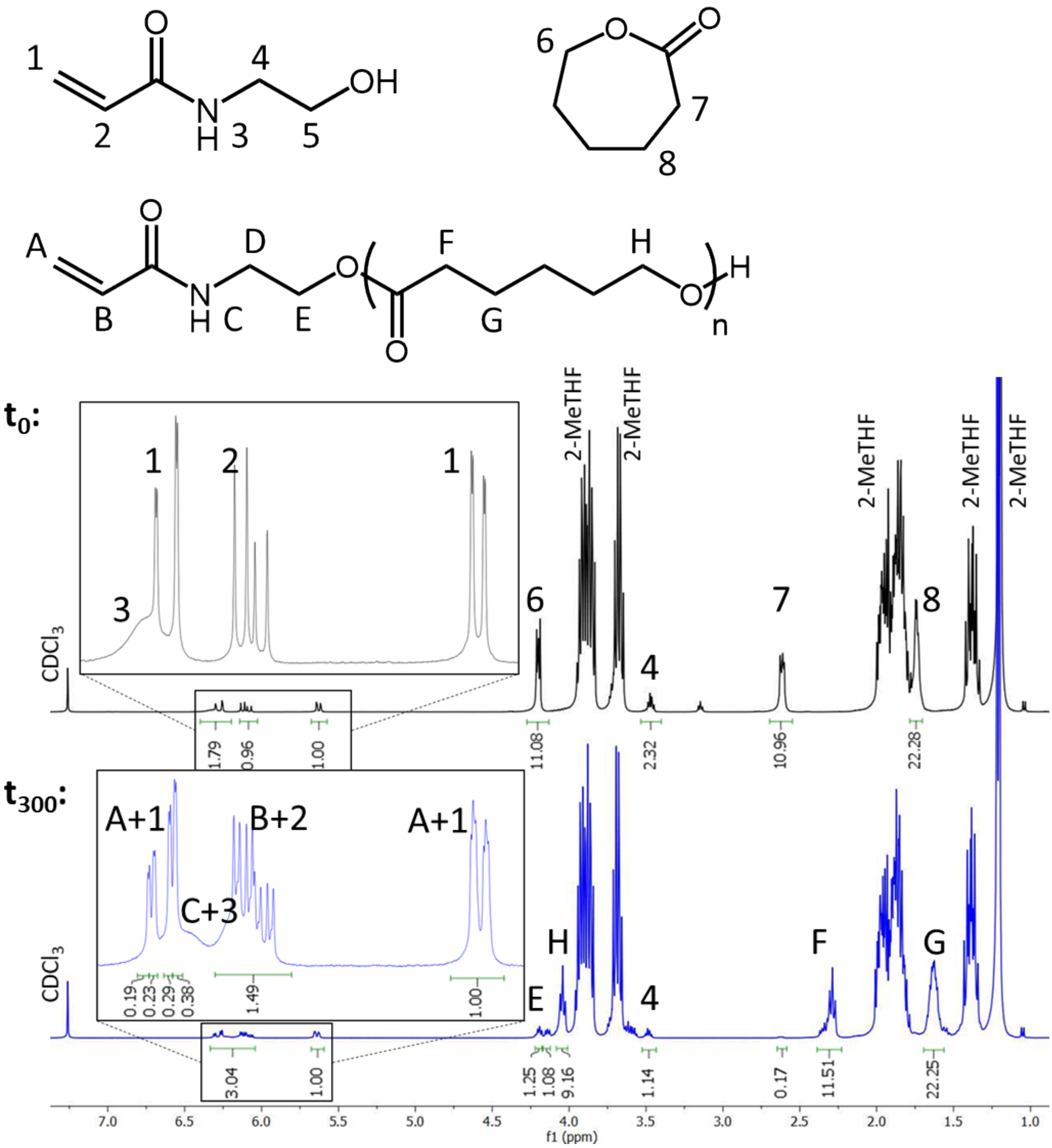

SI Figure 8 ^{1}H-NMR spectra of HEAA initiated eROP of CL (1:5 I:M) in 2-MeTHF (10 wt.% N435) at time=zero (t_0) and time=300 min (t_{300}). No peaks corresponding to HEAA-PCL can be seen at t_0, although at t_{300} unreacted initiator can be detected. 2-MeTHF overlaps with many peaks in the system.

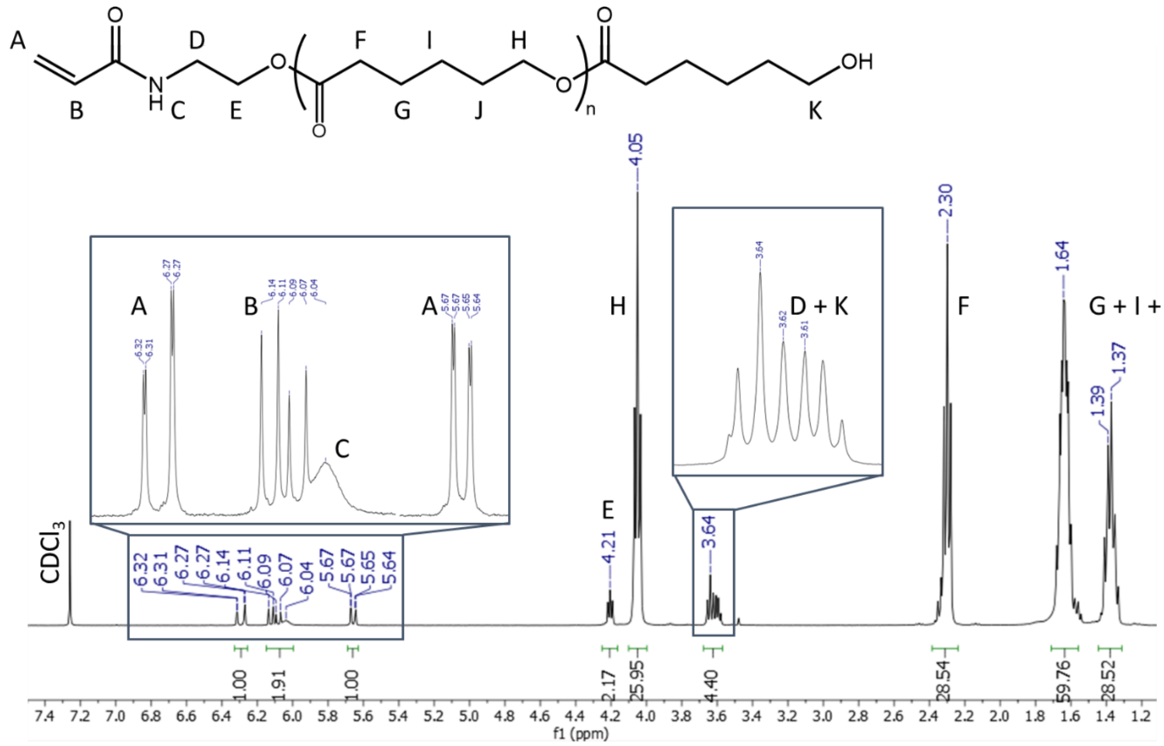

SI Figure 9 ^{1}H-NMR spectrum of HEAA-PCL after precipitation. Peaks H and A were used to determine DP=13, significantly overshooting the targeted DP=5. The inset between 5.4 and 7.0 ppm highlights the region with the olefinic acrylamide peaks.

SI Table 1 Time and conversion of CL and HEAA in 2-MeTHF with 10 wt. % N435, varying the targeted molecular weight by changing the ratio of initiator to monomer.

Time (min)	Conversion of CL into PCL (%)[a]				Conversion of HEAA into HEAA-PCL (%)[a]			
	1:5 HEAA:CL	1:10 HEAA:CL	1:20 HEAA:CL	1:40 HEAA:CL	1:5 HEAA:CL	1:10 HEAA:CL	1:20 HEAA:CL	1:40 HEAA:CL
0	0	0	0	0	0	0	0	0
5	8.48	9.00	7.18	12.2	29.2	31.1	30.1	48.4
15	22.2	22.8	23.5	27.5	40.9	42.9	53.9	62.0
30	40.1	39.5	38.4	46.8	44.3	50.5	60.8	66.8
45	52.0	53.0	50.1	58.4	47.6	54.0	64.9	72.6
60	61.7	62.7	62.6	67.4	50.6	56.5	69.1	72.7
120	83.6	85.8	82.8	89.1	52.0	61.6	72.5	73.5
180	92.8	94.4	93.0	95.8	54.3	61.1	74.2	76.4
240	96.7	97.7	96.6	98.5	52.0	63.1	71.8	76.0
300	98.5	98.9	98.5	99.3	54.1	61.0	73.0	74.4

[a] Determined by H-NMR spectroscopy, following Equation S2 and Equation S3. Monomer concentration of 0.143 gmL^{-1}.

SI Table 2 Molecular mass at various time points for HEAA initiated eROP of CL (I:M 1:20).

Time (min)	Full HEAA M_n($gmol^{-1}$) [a]*	M_n ($gmol^{-1}$) [a]	M_n ($gmol^{-1}$) [b]	M_w ($gmol^{-1}$) [b]	Đ[b]
0	0	0	-	-	-
5	296	718.4	-	-	-
15	708	1214	-	-	-
30	1084	1709	2368	3033	1.28

45	1380	2063	2537	3536	1.39
60	1697	2404	2687	3850	1.43
120	2206	3000	3871	5606	1.45
180	2465	3280	4810	6585	1.37
240	2557	3517	4437	6988	1.58
300	2605	3523	5067	7642	1.51

[a] *Determined by H-NMR spectroscopy, taking HEAA conversion into account following Equation S4 and Equation S5.* [b]*Determined by GPC in THF, calibrated using PMMA standards. "Full HEAA* M_n^{a*}*" was calculated assuming full HEAA conversion. Blank entries were below the limit of calibration. Monomer concentration of 0.143 gmL*$^{-1}$*.*

SI Table 3 Time and conversion of VL and HEAA in 2-MeTHF with 10 wt. % N435, varying the targeted molecular weight by changing the ratio of initiator to monomer.

Time (min)	Conversion of VL into PVL (%)[a]				Conversion of HEAA into HEAA-PVL (%)[a]			
	1:5 HEAA:VL	1:10 HEAA:VL	1:20 HEAA:VL	1:40 HEAA:VL	1:5 HEAA:VL	1:10 HEAA:VL	1:20 HEAA:VL	1:40 HEAA:VL
0	0.0	0.0	0.0	0.0	0.0	0.0	0.0	0.0
5	11.7	11.1	12.6	10.7	19.6	22.8	33.6	32.9
15	20.4	21.9	24.2	21.3	28.6	38.9	50.6	46.7
30	32.1	36.8	38.7	33.4	31.4	51.6	57.8	52.3
45	40.3	46.9	49.7	43.0	35.3	55.0	60.4	56.0
60	49.7	56.1	57.8	51.0	34.9	57.6	62.7	62.0
120	63.8	77.2	79.0	73.6	41.3	63.1	65.6	69.0
180	75.3	84.6	85.4	83.7	47.8	61.7	71.5	71.7
240	81.2	88.0	87.7	87.9	49.1	63.3	68.1	72.0
300	85.2	89.3	88.8	89.5	50.5	65.6	70.7	73.6

[a] *Determined by H-NMR spectroscopy, following Equation S2 and Equation S3. Monomer concentration of 0.143 gmL*$^{-1}$*.*

SI Table 4 Turnover Frequency of lactone monomer during reaction.

Time (min)	Turnover Frequency (µmol min^{-1} g^{-1})[a]							
	1:5 HEAA:CL	1:10 HEAA:CL	1:20 HEAA:CL	1:40 HEAA:CL	1:5 HEAA:VL	1:10 HEAA:VL	1:20 HEAA:VL	1:40 HEAA:VL
0	0	0	0	0	0	0	0	0
5	1487	1570	1744	2147	2339	2216	2510	2147
15	1296	1332	1386	1610	1361	1456	1610	1417
30	1173	1154	1240	1368	1070	1225	1289	1112
45	1013	1033	1085	1138	895	1041	1103	955
60	903	917	964	986	827	934	962	848
120	611	627	636	651	531	643	657	613
180	452	460	464	467	418	470	474	464
240	353	357	359	360	338	366	365	366
300	288	289	290	290	284	297	296	298

[a] *Determined by H-NMR spectroscopy. Monomer concentration of 0.143 gmL*$^{-1}$*.*

MALDI-TOF MS

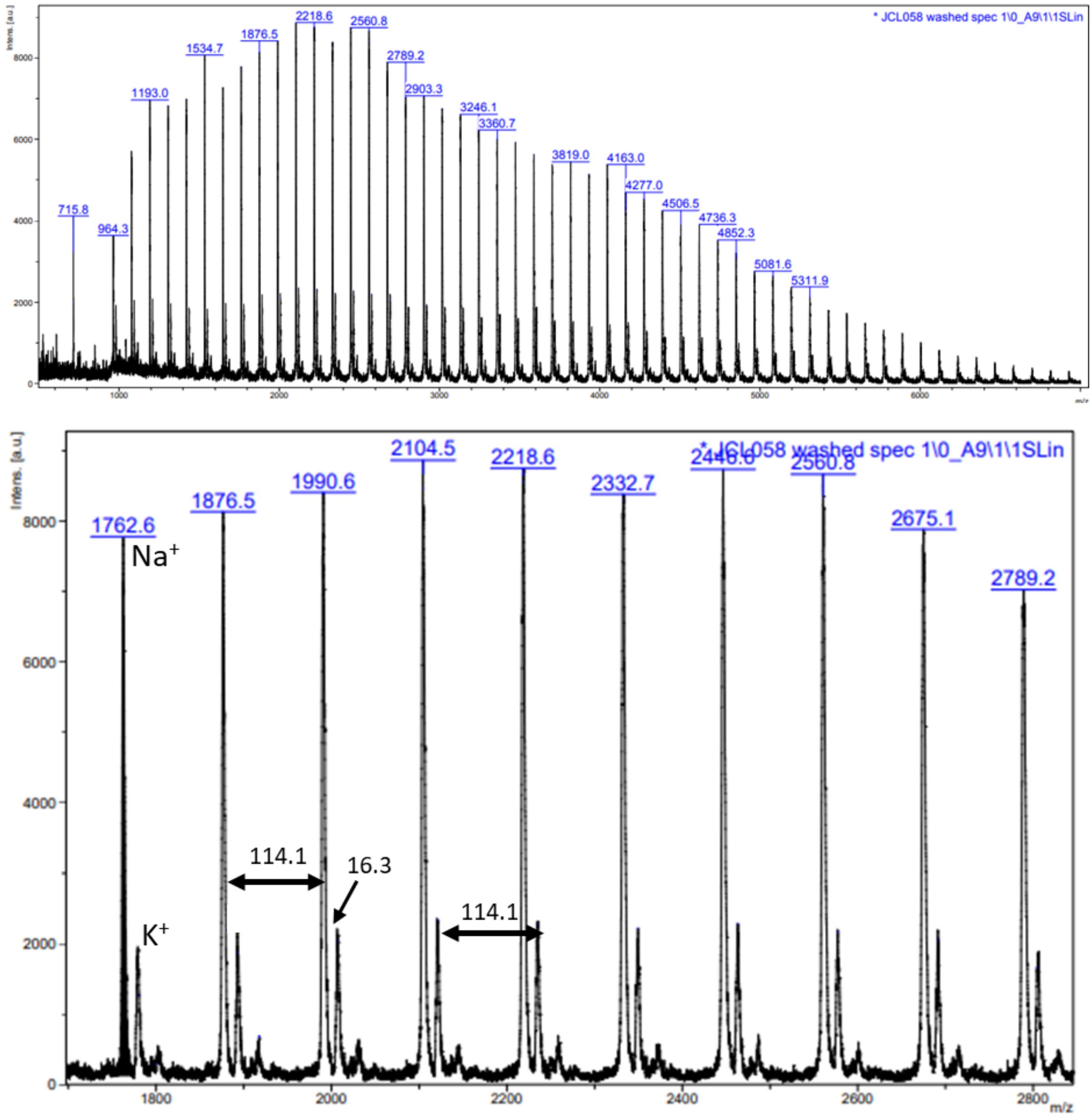

SI Figure 10 MALDI-TOF mass spectrum of HEAA-PCL. Showing an increase in m/z 114.1 between peaks, corresponding to a single unit of caprolactone. Both Na^+ and K^+ counterions can be seen, with a difference in m/z of 16.3. Average M_n by ^{1}H-NMR is 1797 gmol^{-1}.

Control Experiment

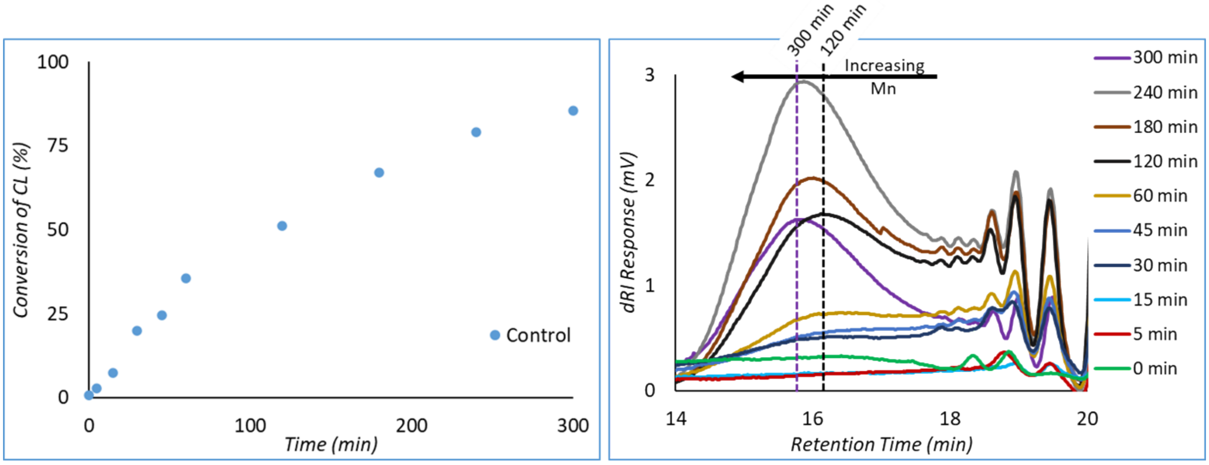

SI Figure 11 Novozym 435 catalysed eROP of CL without explicit addition of initiating species. Left: Conversion determined by ^{1}H-NMR spectroscopy plotted against time. Right: GPC traces during reaction progression. Monomer concentration of 0.143 gmL^{-1}.

SI Table 5 Tabulated data of N435 catalysed eROP without explicit addition of initiating species. [a]

Time (min)	Conversion of CL (%)[a]	M_n (gmol^{-1}) [b]	M_w (gmol^{-1}) [b]	Đ[b]
0	0.8	-	-	-
5	2.6	-	-	-
15	7.3	-	-	-
30	20.0	-	-	-
45	24.5	-	-	-
60	35.5	-	-	-
120	51.2	4954	8685	1.75
180	67.1	4389	8475	1.93
240	79.0	5054	9538	1.89
300	85.5	5147	9686	1.88

[a] Determined by ^{1}H-NMR spectroscopy, [b] determined by GPC. Monomer concentration of 0.143 gmL^{-1}.

Plotting First-Order Kinetics

Ratio of HEAA:Lactone was taken to be constant throughout the reaction, as such HEAA can be used as an internal ^{1}H-NMR standard. The ratio of monomer to initiator/ internal standard, M, was calculated following Equation S6. In the case of HEAA: caprolactone and HEAA: valerolactone this equation takes the forms of Equation S7 and Equation S8 respectively. Using the value of M obtained the natural logarithm of M at time zero (M_0) over M can be taken and subsequently plotted against time. To account for HEAA being consumed both the unreacted and reacted HEAA was incorporated into the internal standard integral.

$$M = \frac{0.5 \int Lactone}{\int Internal\ standard}$$

Equation S6 Ratio of monomer to initiator / internal standard.

$$M = \frac{0.5\int 2.69 - 2.56\ ppm}{\int 6.31 - 6.24\ ppm}$$

Equation S7 Ratio of monomer to initiator / internal standard in the case of caprolactone (monomer) and HEAA (initiator / internal standard).

$$M = \frac{0.5\int 2.60 - 2.48\ ppm}{\int 6.31 - 6.24\ ppm}$$

Equation S8 Ratio of monomer to initiator / internal standard in the case of valerolactone (monomer) and HEAA (initiator / internal standard).

Data obtained for HEAA initiated eROP of CL and VL following Equation S6 can be seen in SI Table 5 and SI Table 6.

SI Table 6 Tabulated data used to plot first-order kinetics for HEAA initiated eROP of CL (2-MeTHF, 65 °C, 10 wt.% N435). M_0 designates the value of M at time zero.

Time (min)	1:5 HEAA:CL		1:10 HEAA:CL		1:20 HEAA:CL		1:40 HEAA:CL	
	M	$\ln(M_0/M)$	M	$\ln(M_0/M)$	M	$\ln(M_0/M)$	M	$\ln(M_0/M)$
0	6.07	0.00	11.81	0.00	23.38	0.00	39.12	0.00
5	4.61	0.27	9.37	0.23	17.77	0.27	33.94	0.14
15	3.69	0.50	7.14	0.50	15.97	0.38	28.91	0.30
30	3.20	0.64	6.38	0.62	13.03	0.59	26.28	0.40
45	2.53	0.88	4.94	0.87	10.02	0.85	20.58	0.64
60	2.07	1.08	3.90	1.11	7.54	1.13	14.51	0.99
120	0.88	1.93	1.79	1.89	3.48	1.91	5.13	2.03
180	0.49	2.52	0.64	2.91	1.29	2.90	2.21	2.88
240	0.17	3.57	0.31	3.64	0.53	3.79	0.83	3.85
300	0.09	4.21	0.12	4.61	0.23	4.62	0.37	4.66

Monomer concentration of 0.143 gmL^{-1}.

SI Table 7 Tabulated data used to plot first-order kinetics for HEAA initiated eROP of VL (2-MeTHF, 65 °C, 10 wt.% N435). M_0 designates the value of M at time zero.

Time (min)	1:5 HEAA:VL		1:10 HEAA:VL		1:20 HEAA:VL		1:40 HEAA:VL	
	M	$\ln(M_0/M)$	M	$\ln(M_0/M)$	M	$\ln(M_0/M)$	M	$\ln(M_0/M)$
0	5.34	0.00	10.78	0.00	22.85	0.00	49.74	0.00
5	4.37	0.20	9.89	0.09	18.82	0.19	44.94	0.10
15	4.15	0.25	8.24	0.27	15.33	0.40	39.53	0.23
30	3.22	0.51	7.14	0.41	13.31	0.54	35.46	0.34
45	3.36	0.46	5.18	0.73	12.50	0.60	28.88	0.54
60	2.70	0.68	5.18	0.73	9.28	0.90	29.84	0.51
120	2.15	0.91	2.51	1.46	4.55	1.61	15.39	1.17
180	1.30	1.42	1.79	1.79	3.38	1.91	9.98	1.61
240	0.92	1.75	1.21	2.18	2.85	2.08	7.10	1.95
300	0.81	1.89	1.19	2.20	2.66	2.15	6.70	2.00

Monomer concentration of 0.143 gmL^{-1}.

Deeper Kinetic Analysis

SI Table 7 shows the kinetic constant (*k*) associated with monomer conversion for different initiator:monomer ratios based on the first-order kinetic model. The analytical solution for a first-order model (Equation S10) expressed in terms of monomer conversion (Equation S9) successfully describes the conversion profiles of caprolactone throughout enzymatic reaction (SI Figure 12).

$$x = 1 - e^{(-k \cdot t)}$$

Equation S9

$$\frac{dM}{dt} = -k \cdot M$$

Equation S10

$$\frac{dM}{dt} = -k \cdot M^2$$

Equation S11

$$x = \frac{M_0 \cdot k \cdot t}{1 + M_0 \cdot k \cdot t}$$

Equation S12 Where M is the monomer concentration, M_0 is the initial monomer concentration, t corresponds to reaction time and k stands for the kinetic constant associated to monomer consumption throughout enzymatic reaction. Where x corresponds to the dimensionless conversion, k is the first-order kinetic constant, and t is the reaction time.

Considering the similar behaviour of monomer conversion (SI Figure 12), it is crucial to evaluate if the estimated constants can be considered different from a statistical point of view. According to SI Figure 13A, we can assume that the kinetic constants are statistically different with a level of confidence of 95.0%, except for a particular comparison of *k* estimated for 1:5 HEAA:CL and 1:10 HEAA:CL conditions, where it is not reasonable to state unequivocally that the estimated parameters are statistically different. As an additional and important piece of information, SI Figure 13A shows that the estimated parameter *k* can be expressed as a linear function ($k=1.05\cdot10^{-4}$ (HEAA:CL)+0.015611, $R^2=0.991$) of the caprolactone units (HEAA:CL) added to the polymerisation system at the beginning of the enzymatic reaction. On the other hand, when both Equation S9 and statistical inference are applied to VL, the experimental data are not well explained by the first-order model (SI Figure 14). Equation S9 also overestimates the monomer conversion after 120 min reaction time independent of the HEAA:VL ratio evaluated. A comparative analysis of the estimated values for the kinetic constants (SI Figure 15A) strongly indicates that, from a statistical point of view only, the value of *k* associated with the 1:5 HEAA:VL condition may be considered different from the one related to 1:20 HEAA:VL. Additionally, (SI Figure 15A), a linear relation between *k* and the initiator: monomer ratio cannot be established, as a result of the kinetic behaviour of the enzymatic reaction carried out with 1:40 HEAA:VL, exhibiting in the first hour of reaction a conversion profile very similar to the reaction performed at a 1:5 HEAA:VL ratio. Alternatively, a second-order kinetic model can be used to describe the conversion profile of VL, as illustrated in SI Figure 16. According to the model predictions (Equations S11 and S12), the second-

order model fits better to the VL conversion data, but it loses reliability in predicting conversion within the first 60 minutes of reaction.

SI Table 8 Kinetic constants for first-order monomer consumption.

I:M	Kinetic constant, k (min^{-1}) [a]	
	HEAA:CL	HEAA:VL
1:5	0.0161 ± 0.0003	0.0093 ± 0.0009
1:10	0.0166 ± 0.0001	0.0130 ± 0.0009
1:20	0.0179 ± 0.0002	0.0140 ± 0.0012
1:40	0.0197 ± 0.0005	0.0115 ± 0.0007

[a] The kinetic constant expressed as k ± parameter standard error was estimated by using the Levenberg-Marquardt algorithm.

Reactivity of lactones in the presence of N435 is known to increase with ring size.[1] Kinetic constants for VL were found to be significantly lower in comparison than those of CL (SI Table 7), demonstrating lower reactivity in the studied reactions. Discussed in the main text as a result of differing ring-strain. Regardless, both VL and CL were able to be ring-opened enzymatically using HEAA and N435 in the observed timeframe.

Model Prediction

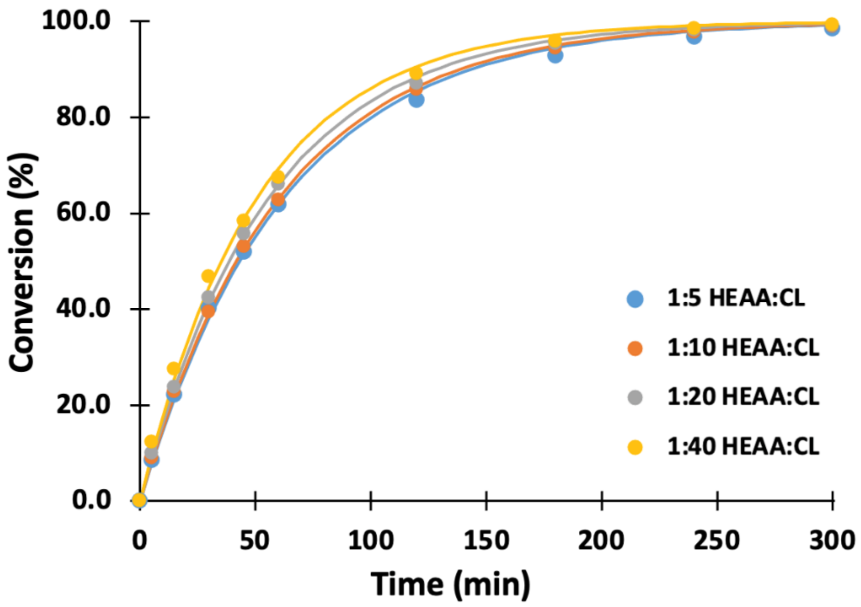

SI Figure 12 First-order model prediction of conversion profiles of caprolactone during enzymatic reaction (N.B. Continuous lines correspond to polymerisation model output.)

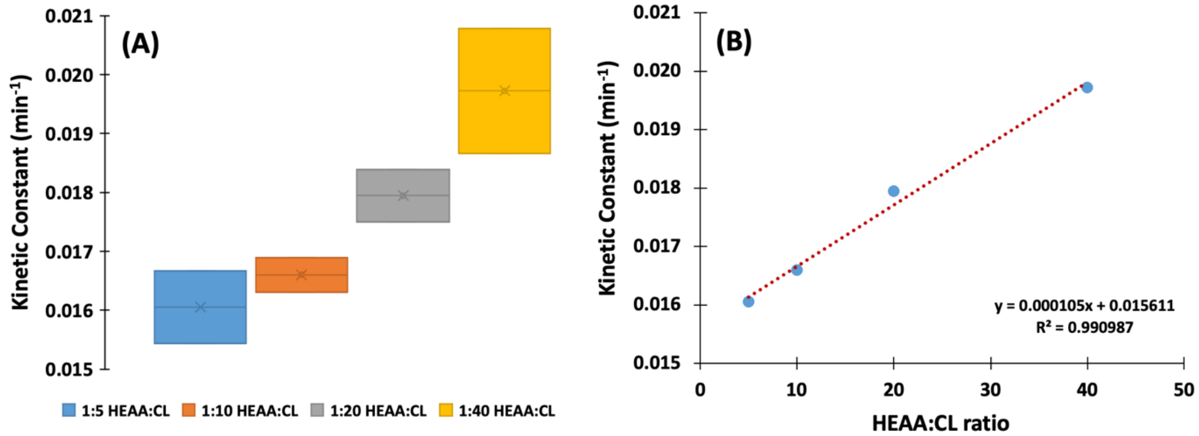

SI Figure 13 . (A) Statistical evaluation of the estimated kinetic constants considering the low and upper limits computed with a confidence a level of confidence: 95.0% (p = 0.050), and (B) Linear dependence of the kinetic constant with respect to HEAA:CL ratio.

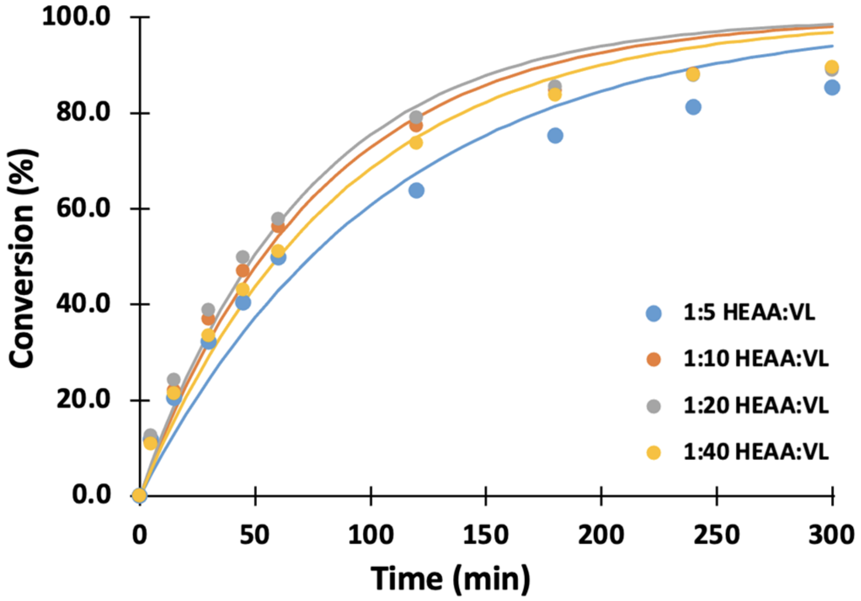

SI Figure 14 First-order model prediction of conversion profiles of valerolactone during enzymatic reaction (N.B. Continuous lines correspond to polymerisation model output.)

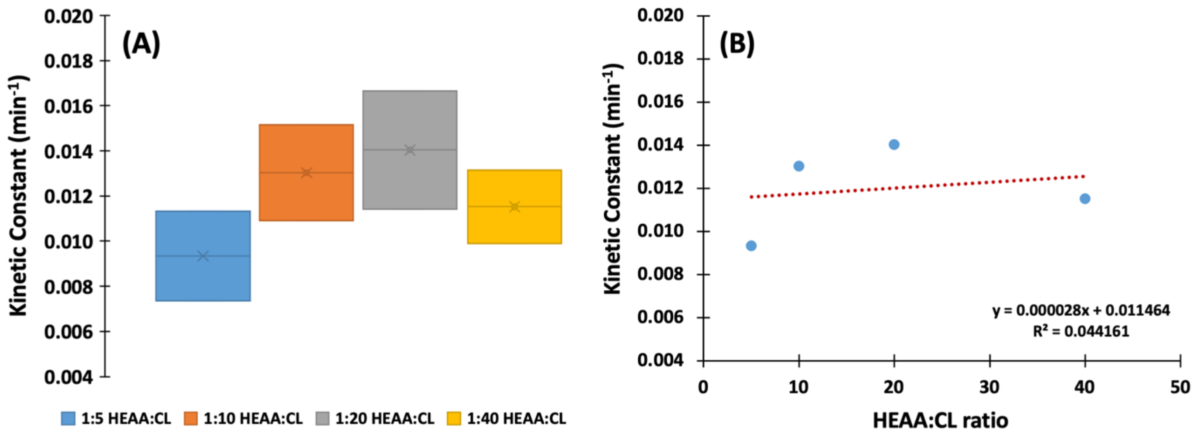

SI Figure 15 (A) Statistical evaluation of the estimated kinetic constants considering the low and upper limits computed with a confidence a level of confidence: 95.0% (p = 0.050), and (B) Linear dependence of the kinetic constant with respect to HEAA:VL ratio.

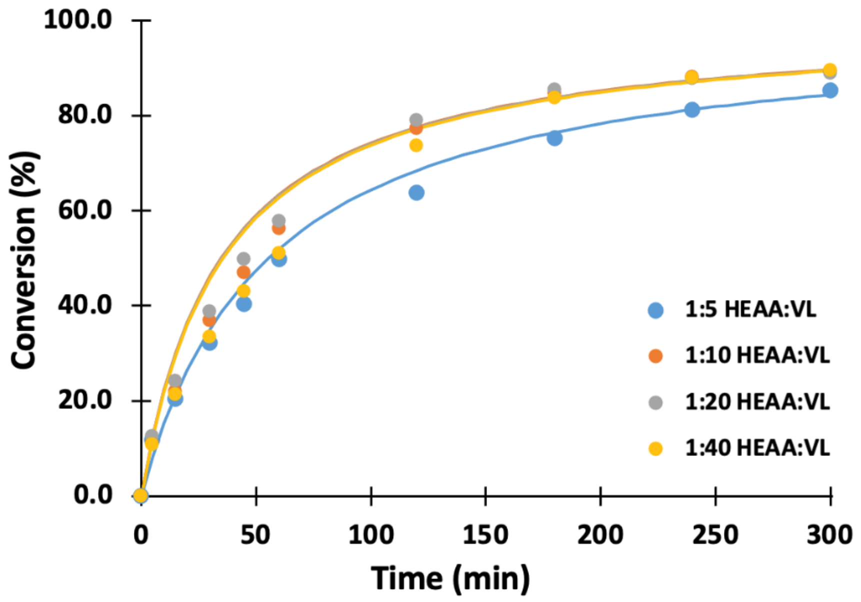

SI Figure 16 Second-order model prediction of the conversion profiles of valerolactone during enzymatic reaction (N.B. Continuous lines correspond to polymerisation model output.)

Mechanism of Monomer Release from Acyl-Enzyme Intermediate

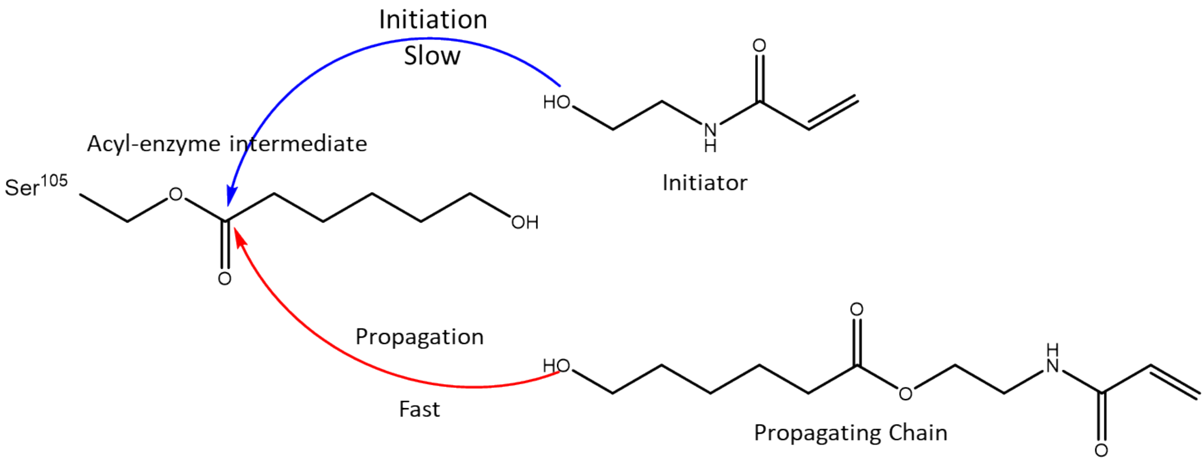

SI Figure 17 The experimental and computational results both suggest propagation is faster than initiation in liberating the activated caprolactone unit from the acyl-enzyme intermediate. This is thought to be a result of the greater binding affinity of the propagating chain to the enzyme active site.

Lactide Extension HEAA-PCL

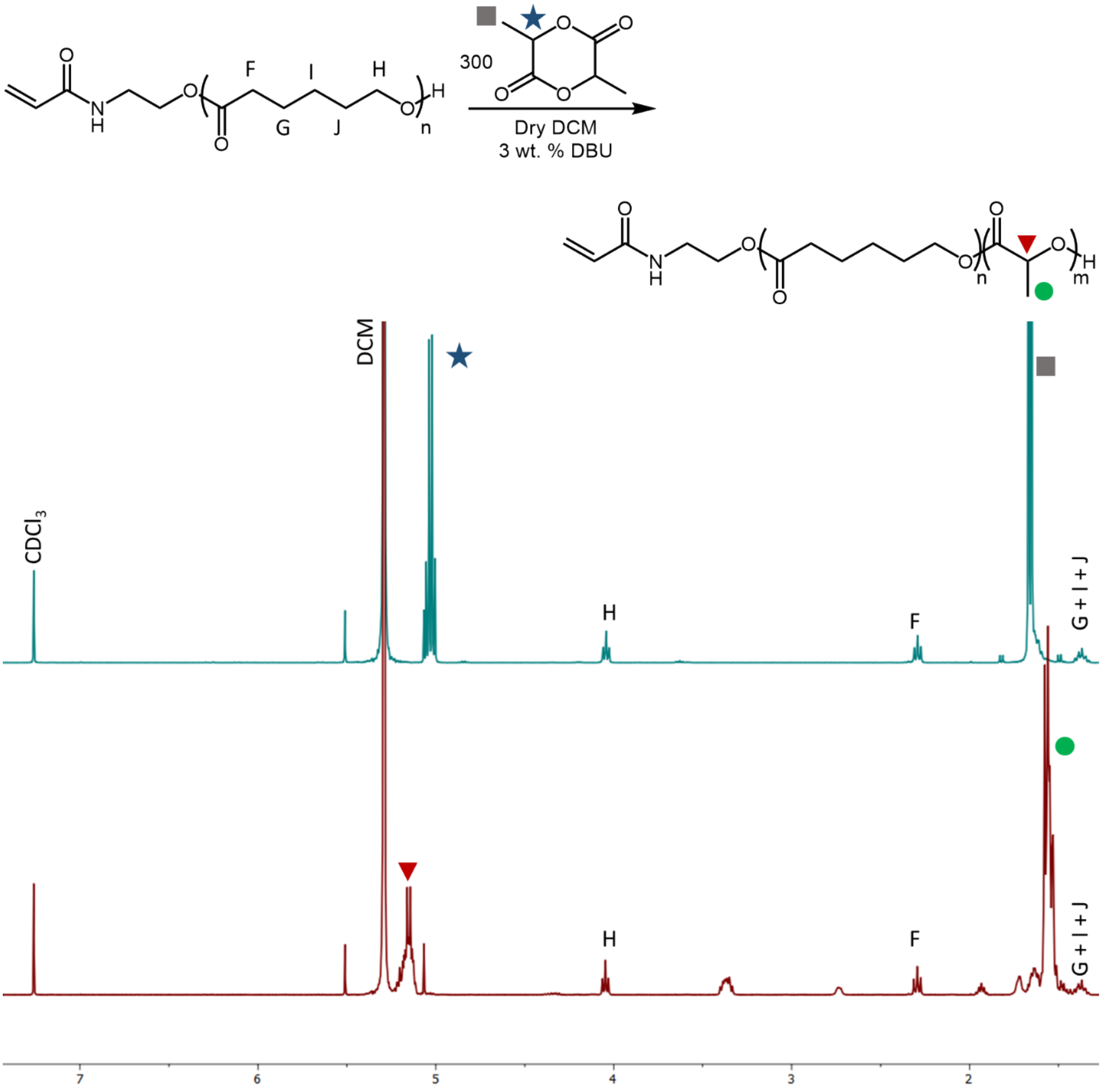

SI Figure 18 ^{1}H-NMR spectra of lactide extension of HEAA-PCL. Top: reaction at time zero. Bottom: reaction at time = 30 minutes.

Recyclability of N435 in 2-MeTHF

Enzyme recyclability is key from cost saving and green chemistry perspectives. The stability of N435 was investigated by repeatedly subjecting the same enzyme beads to reaction conditions, only replacing the liquid phase of the reaction with fresh monomer, initiator, and solvent between cycles. Three experiments were run in parallel, and the average (mean) conversion is plotted in SI Figure 19. Only a minimal decrease in the conversion of CL is detectable by ^{1}H-NMR, dropping from 94.8% for

virgin N435 to 93.2% during the tenth recycle. This decrease of 1.6 % is minimal, and this decrease is still within the acceptable error for ^{1}H-NMR. From literature N435 has been recycled up to 15 times with minimal decrease in reactivity, varying with temperature and solvent used during the reaction.[2,3] It is expected for N435 to lose activity with each recycle due to factors such as: enzyme leeching into the reaction media, removal of essential water, support degradation, amongst other factors.[3,4]

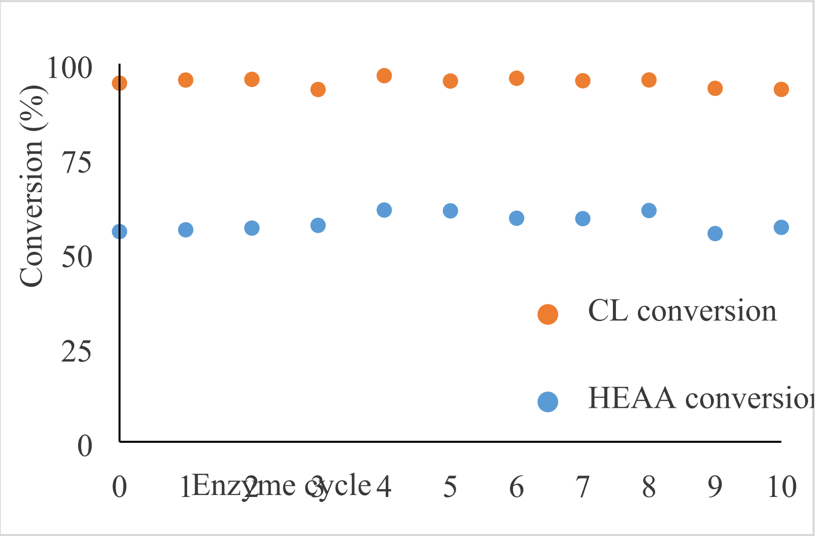

SI Figure 19 Mean percentage conversion of CL and HEAA after 5 h of reaction (65 °C, 2-MeTHF, 10 wt.% N435) while recycling the N435 beads, experiments run in triplicate. Monomer concentration of 0.143 gmL^{-1}.

The data presented here demonstrates that the N435 is a robust enzymatic catalyst capable of being reused up to ten times in the reaction conditions employed without reduction in catalytic activity. CL and HEAA conversion are consistent throughout the enzyme recycles and the maintained activity also demonstrates the suitability of 2-MeTHF as a solvent for N435 catalysed reactions. The excellent recyclability of the immobilized catalyst demonstrated here significantly contributes to the economic and environmental credentials of the reactions.

SI Table 9 Tabulated results of the N435 recyclability study seen in SI Figure 19.

Enzyme recycle	Average HEAA conversion[ab]	σ HEAA conversion[c]	Average CL conversion[ab]	σ CL conversion[c]
0	55.6	0.56	94.8	1.60
1	56.1	0.16	95.7	1.44
2	56.5	0.76	95.9	1.40
3	57.2	0.90	93.2	2.67
4	61.3	0.08	96.8	1.36
5	61.1	1.21	95.4	2.43
6	59.1	1.24	96.2	1.40
7	59.0	0.32	95.5	1.97
8	61.2	0.92	95.7	0.36
9	55.1	2.67	93.5	1.99
10	56.7	2.79	93.2	0.97

[a] Determined by H-NMR spectroscopy, following Equation S2 and Equation S3. [b] Calculated using a simple mean of three reactions run in parallel to ensure reproducibility. [c] Calculated using the STDEV.P function in Microsoft Excel 2016.

Some error in the HEAA conversion can be attributed to the N*H* signal shifting due to hydrogen bonding effects. As a result, some integrations could potentially lose accuracy, see SI Figure 20.

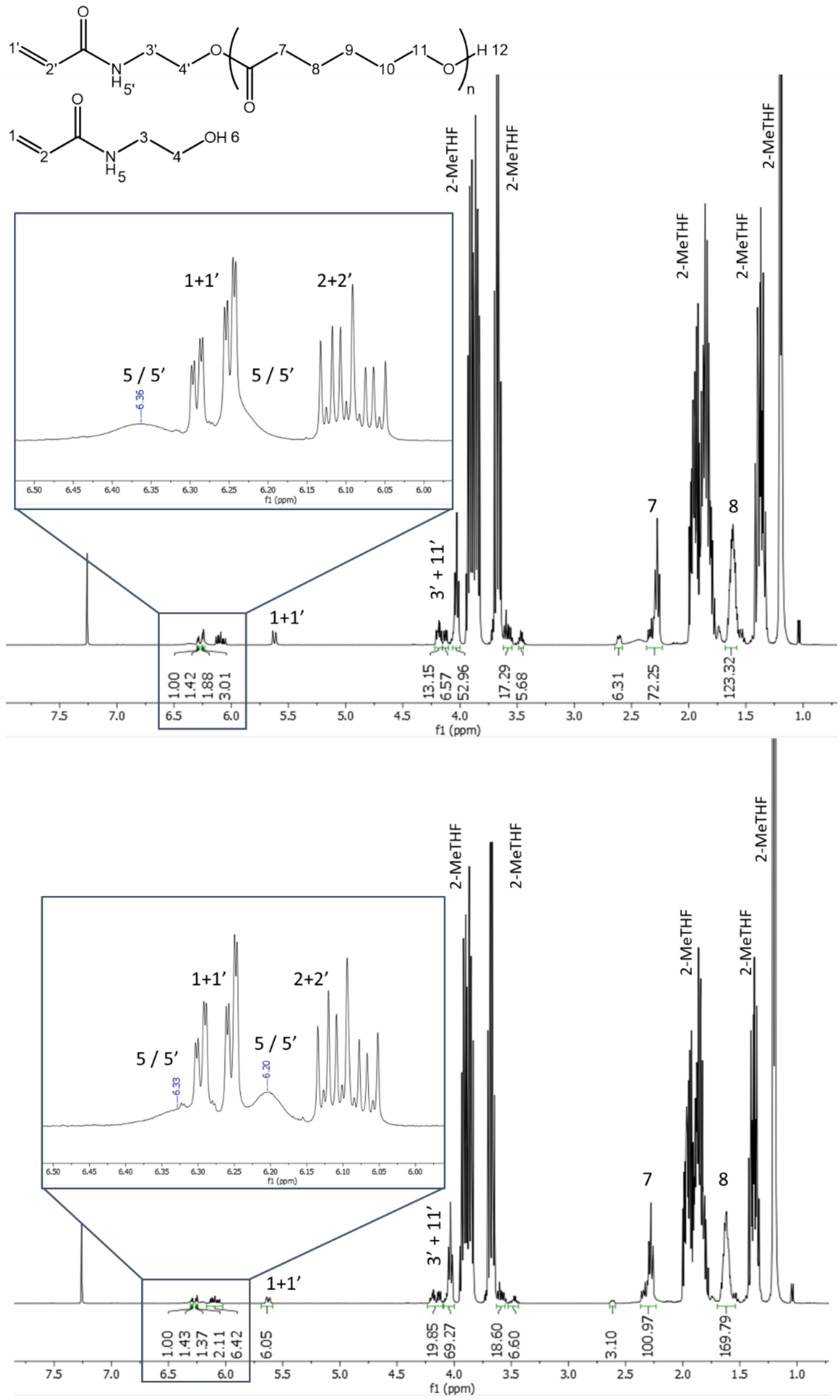

SI Figure 20 Protons 5 and 5' can shift and potentially introduce error into HEAA conversion calculations. Both top and bottom are ^{1}*H NMR spectra collected during enzyme recycling studies in 2-MeTHF after cycle 5, collected from two separate reactions running in parallel. Note that the plethora of peaks corresponding to 2-MeTHF covers several peaks.*

Alternative initiator Species

N-Hydroxyethyl methacrylamide (HEMAM) was synthesised, following a known procedure,[5] to observe the effect of increasing initiator hydrophobicity. It was hypothesised that an increase in hydrophobicity would lead to an improvement in initiator conversion by increasing the affinity for the active site of CALB. It was found however that HEMAM and HEAA had similar reactivity in eROP of CL. CL conversion reaches 99.7% conversion, HEMAM conversion reaches a conversion of 66.9% (SI Figure 21). The final initiator conversion reached does not vary significantly between HEMAM and HEAA.

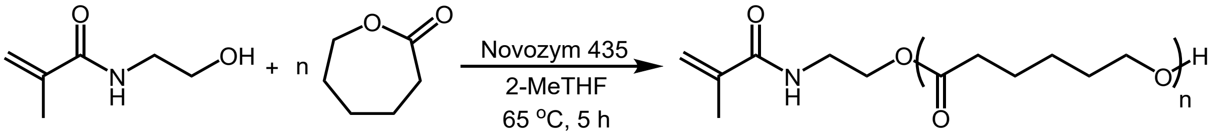

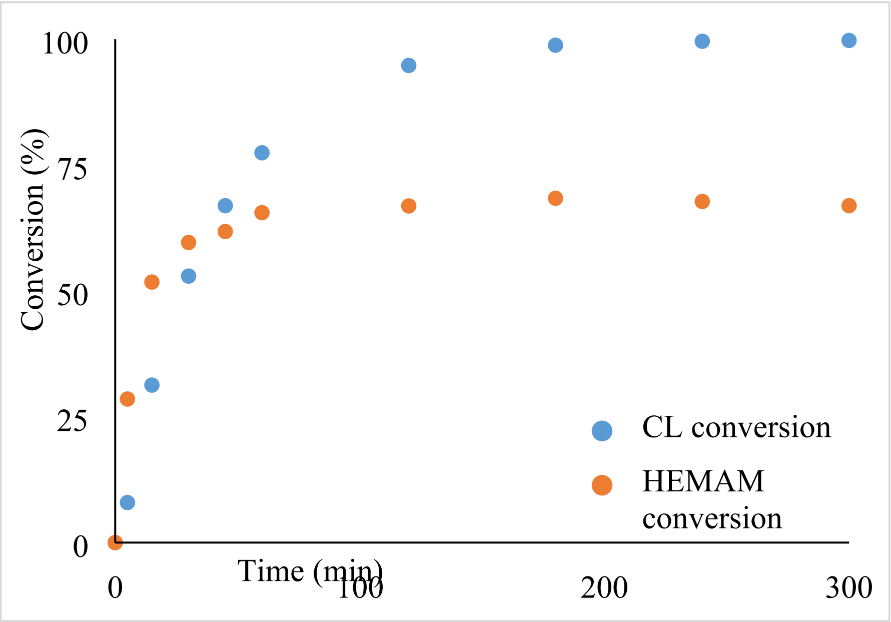

SI Figure 21 Top: Reaction scheme. Bottom: Conversion of HEMAM and CL in the HEMAM initiated eROP of CL in 2-MeTHF using 10 wt. % N435. Monomer concentration of 0.143 gmL^{-1}.

These experimental findings were also backed up by computational docking studies, which found that HEAA and HEMAM have very similar affinities for the active site of CALB (SI Table 9), explaining the very similar behaviour observed.

SI Table 10 Autodock and GBSA results of HEMAM, HEMAM-CL, HEAA, and HEAA-CL in the active site of CALB.

Structure	Autodock Score (kcal/mol)	GBSA Binding Energy (kcal/mol)
HEMAM	-4.31	-19.90
HEMAM-CL	-5.58	-31.18
HEAA	-4.31	-16.04
HEAA-CL	-5.50	-32.75

Varying enzyme loadings for HEAA initiated eROP of CL

HEAA initiated eROP of CL in 2-MeTHF was performed using a variety of enzyme loadings to investigate if altering the rate of reaction would alter the final conversion of HEAA into HEAA-PCL. From SI Table 10 it can be seen that quantitative conversion of CL into PCL was achieved for all enzyme loadings after 17 h. The conversion of HEAA obtained did not vary significantly with increased catalyst loading.

SI Table 11 Conversion of CL and HEAA after 17 h in 2-MeTHF at 65°c, varying loadings of N435. For 0 wt. % N435 no peaks corresponding to HEAA-PCL could be seen.

Enzyme loading (wt.% w.r.t. CL)	Conversion of CL into PCL (%)[a]			Conversion of HEAA into HEAA-PCL (%)[a]		
	1:5 HEAA:CL	1:10 HEAA:CL	1:25 HEAA:CL	1:5 HEAA:CL	1:10 HEAA:CL	1:25 HEAA:CL
0	0	0	0	0	0	0
5	99.2	99.8	99.8	55.7	64.0	70.4
10	99.8	99.8	99.8	55.9	64.4	69.7
20	99.8	99.8	99.8	56.4	59.9	70.9

[a] *Determined by H-NMR spectroscopy, following Equation S2 and Equation S3.*

GPC traces

SI Table 12 Data obtained for a series of polymers and macromonomers.

Polymer	Ratio (HEAA-PCL): Comonomer	Conversion (%)[a]	Mn GPC (kDa) [b]	Mw GPC (kDa) [b]	Đ [b]
Poly-(HEAA-PCL)$_{44}$	1:0	Quant.	14.1	21.9	1.6
(HEAA-PCL)$_{44}$-*co*-PEGMA	4:6	99	5.39	9.66	1.8
(HEAA-PCL)$_{44}$-*co*-PEGMA	7:3	94	11.2	15.8	1.4
Poly-(HEAA-PCL)$_{15}$	1:0	Quant.	9.18	13.2	1.4
(HEAA-PCL)$_{15}$-*co*-PEGMA	4:6	98	4.00	5.36	1.3
(HEAA-PCL)$_{15}$-*co*-PEGMA	7:3	99	5.71	8.87	1.6
(HEAA-PCL)$_{10}$-*co*-HEAA	Approx. 1.8:0.17	Quant.	8.90	15.4	1.7
(HEAA-PCL)$_{15}$ macromonomer	-	-	3.13	3.90	1.2
(HEAA-PCL)$_{44}$ macromonomer	-	-	9.89	13.2	1.3

[a] Determined by ^{1}H-NMR spectroscopy, [b] determined by GPC.

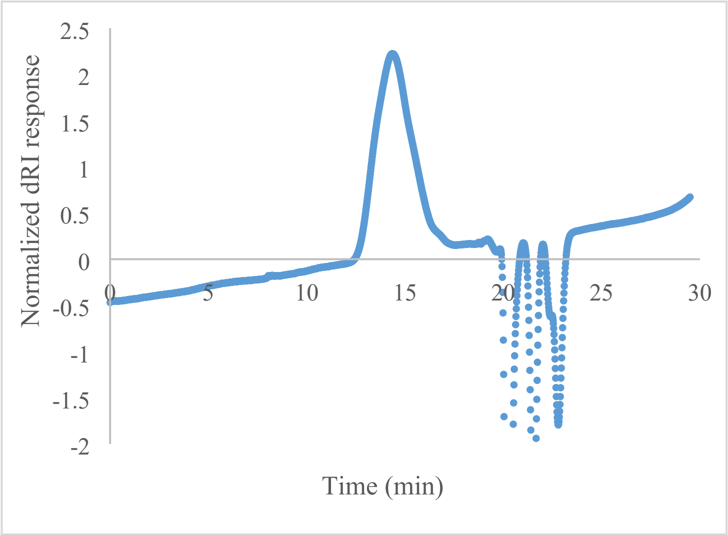

SI Figure 22 GPC of Poly-(HEAA-PCL)$_{44}$.

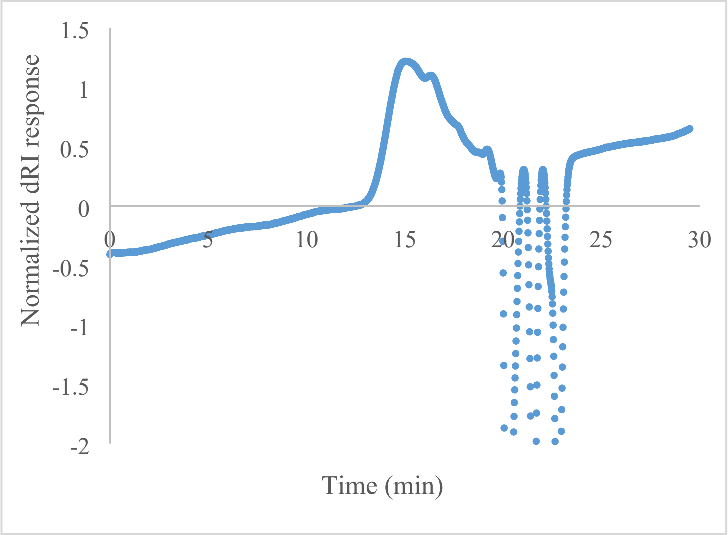

*SI Figure 23 GPC of (HEAA-PCL)$_{44}$-*co*-PEGMA 4:6.*

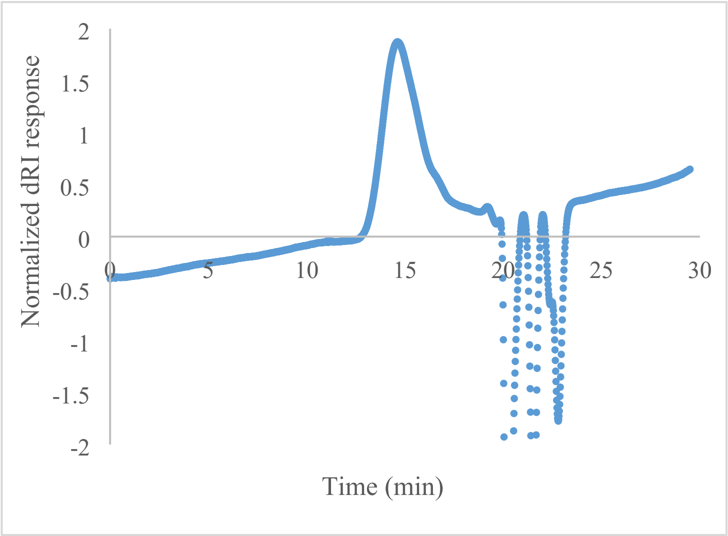

*SI Figure 24 GPC of (HEAA-PCL)$_{44}$-*co*-PEGMA 7:3.*

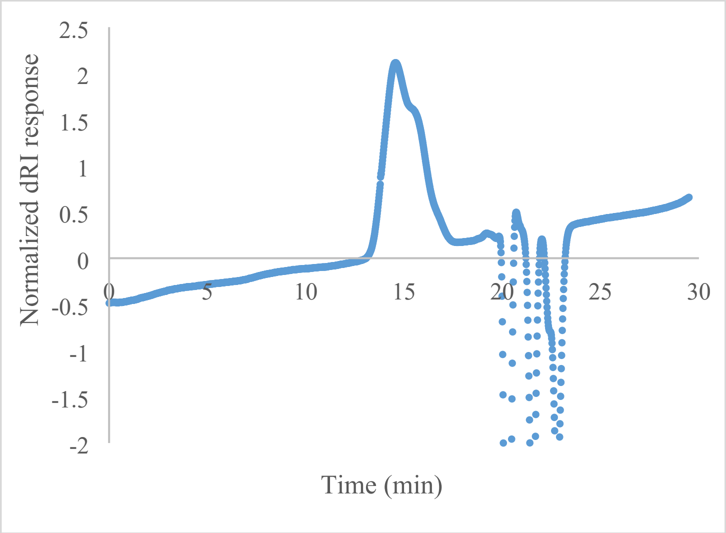

SI Figure 25 GPC of Poly-(HEAA-PCL)$_{15}$.

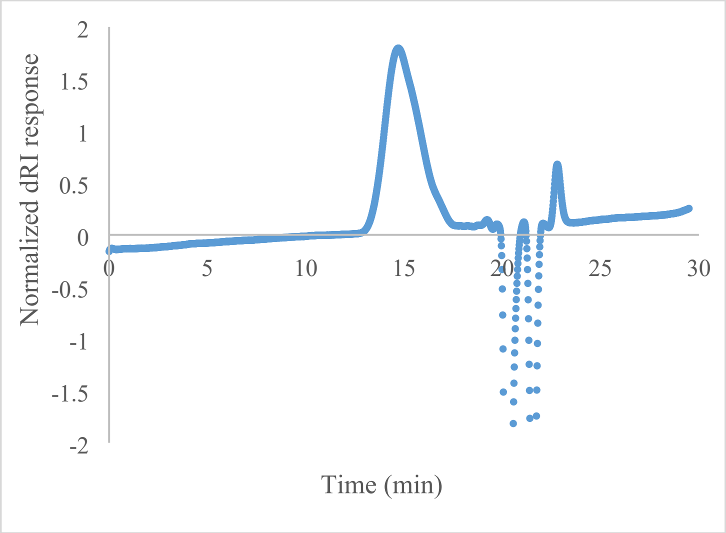

SI Figure 26 (HEAA-PCL)$_{44}$ macromonomer.

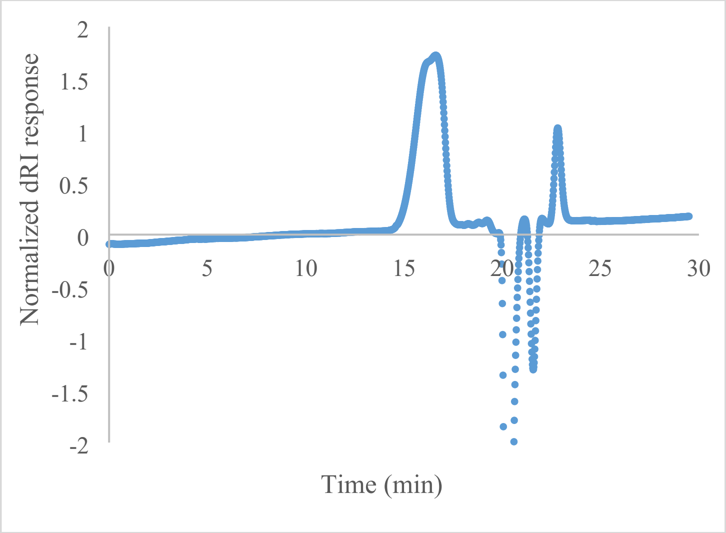

SI Figure 27 (HEAA-PCL)$_{15}$ macromonomer.

DLS Plots

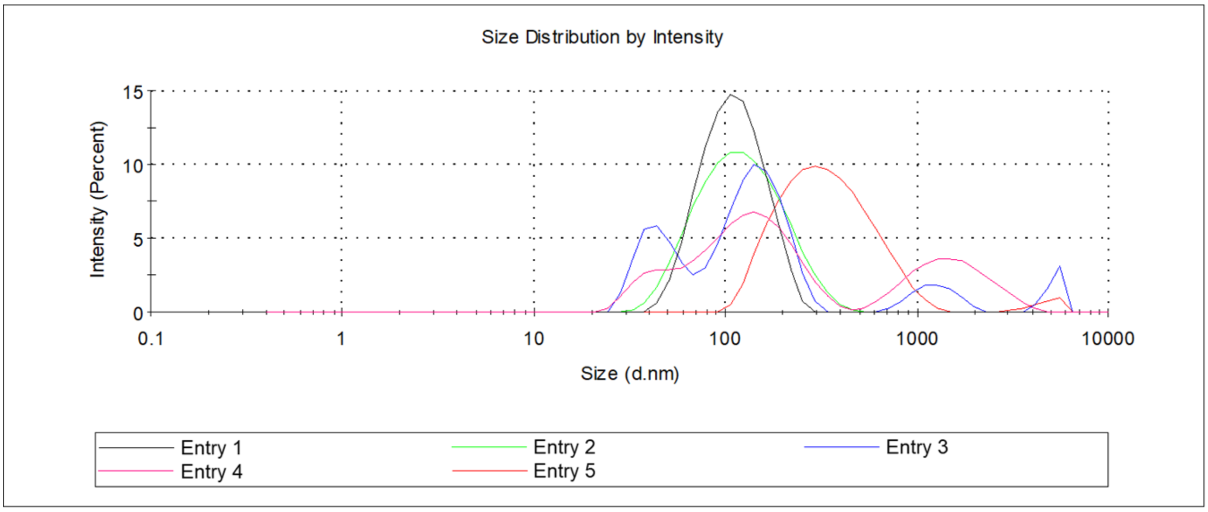

SI Figure 28 DLS Plots of polymeric nanoparticles in water (1 mgmL^{-1}). "Entry" codes refer to Table 3 of the main text.

Sources

1 A. Duda, A. Kowalski, S. Penczek, H. Uyama and S. Kobayashi, *Macromolecules*, 2002, **35**, 4266–4270.

2 M. M. R. Talukder, J. C. Wu, S. K. Lau, L. C. Cui, G. Shimin and A. Lim, *Energy and Fuels*, 2009, **23**, 1–4.

3 A. Pellis, L. Corici, L. Sinigoi, N. D'Amelio, D. Fattor, V. Ferrario, C. Ebert and L. Gardossi, *Green Chem.*, 2015, **17**, 1756–1766.

4 F. Hollmann, P. Grzebyk, V. Heinrichs, K. Doderer and O. Thum, *J. Mol. Catal. B Enzym.*, 2009, **57**, 257–261.

5 G. Y. N. Chan, M. G. Looney, D. H. Solomon and S. Veluayitham, *The Synthesis of Novel Hybrid Monomers*, 1998, vol. 51.
